# Kingianic Acids A–G, Endiandric Acid Analogues from *Endiandra kingiana*

**DOI:** 10.3390/molecules19021732

**Published:** 2014-01-31

**Authors:** Mohamad Nurul Azmi, Charlotte Gény, Aurélie Leverrier, Marc Litaudon, Vincent Dumontet, Nicolas Birlirakis, Françoise Guéritte, Kok Hoong Leong, Siti Nadiah Abd. Halim, Khalit Mohamad, Khalijah Awang

**Affiliations:** 1 Department of Chemistry, Faculty of Science, University Malaya, Kuala Lumpur 50603, Malaysia; E-Mails: libra_mine85@yahoo.co.uk (M.N.A.); nadiahhalim@um.edu.my (S.N.A.H.); 2 Centre de Recherche de Gif, Institut de Chimie des Substances Naturelles (ICSN), CNRS, Labex LERMIT, Gif-sur-Yvette Cedex 91198, France; E-Mails: charlotte.geny@cnrs.fr (C.G.); aurelie.leverrier@gmail.com (A.L.); vincent.dumontet@cnrs.fr (V.D.); nicolas.birlirakis@cnrs.fr (N.B.); francoise.gueritte@wanadoo.fr (F.G.); 3 Department of Pharmacy, Faculty of Medicine, University Malaya, Kuala Lumpur 50603, Malaysia; E-Mails: Leongkh@um.edu.my (K.H.L.); khalitmohamad@um.edu.my (K.M.)

**Keywords:** *Endiandra kingiana*, lauraceae, endiandric acids, kingianic acids, anti-apoptotic proteins, Bcl-xL, Mcl-1

## Abstract

A phytochemical investigation of the methanolic extract of the bark of *Endiandra kingiana* led to the isolation of seven new tetracyclic endiandric acid analogues, kingianic acids A–G (**1**–**7**), together with endiandric acid M (**8**), tsangibeilin B (**9**) and endiandric acid (**10**). Their structures were determined by 1D- and 2D-NMR analysis in combination with HRMS experiments. The structure of compounds **9** and **10** were confirmed by single-crystal X-ray diffraction analysis. These compounds were screened for Bcl-xL and Mcl-1 binding affinities and cytotoxic activity on various cancer cell lines. Compound **5** showed moderate cytotoxic activity against human colorectal adeno-carcinoma (HT-29) and lung adenocarcinoma epithelial (A549) cell lines, with IC_50_ values in the range 15–17 µM, and compounds **3**, **6** and **9** exhibited weak binding affinity for the anti-apoptotic protein Mcl-1.

## 1. Introduction

In our pursuit to discover bioactive phytochemicals from the Malaysia flora [1,2,3] we recently reported a series of new natural pentacyclic polyketides, named kingianins A-L, isolated from the ethyl acetate extract of the bark of *Endiandra kingiana* Gamble (Lauraceae) [4]. Several kingianins showed strong binding affinity to the anti-apoptotic protein Bcl-xL, and can therefore be considered as potential anticancer agents [4]. In order to discover additional members of this chemical series or close analogues, we have embarked on the investigation on the methanolic extract of the bark of this species. *E. kingiana* is a medium-sized evergreen tree, distributed throughout Peninsular Malaysia and Borneo [5,6,7]. There are about 125 *Endiandra* species found throughout the tropical regions, including 10 species in Malaysia [5,6,7], but to our knowledge only three species: *E. introrsa*, *E. anthropophagorum* and *E. kingiana*, have been studied for their phytochemicals. The first one has been reported to produce interesting cyclic polyketides, named endiandric acids, possessing eight chiral centers, and usually isolated as racemic mixtures [8,9,10,11]. It was postulated by Black and co-workers that they could be formed by non-enzymatic cyclizations (8πe and 6πe electrocyclization followed by Diels-Alder reaction) of a phenylpolyene acid precursor [12,13,14]. In 1982, Nicolaou’s group successfully synthesized the natural endiandric acids by implementing a biomimetic strategy based on Black’s hypothesis [14,15,16,17]. Endiandric acids and their close derivatives, beilschmiedic acids, are the most characteristic type of natural products isolated from *Beilschmiedia* and *Endiandra* species. They were found to exhibit various biological activities, such as antibacterial [18,19,20], antiplasmodial [20], antitubercular [21], iNOS inhibitory activity [22], and anticancer properties [20,23]. Recently, Williams *et al.* reported the cytotoxic and antibacterial activities of a series of beilschmiedic acids isolated from a Gabonese *Beilschmiedia* species against NCI-H460 human lung cancer cells and a clinical isolate of methicillin-resistant *Staphylococcus aureus*, respectively [23]. Talonsti *et al.* have also reported recently the isolation of four beilschmiedic acid derivatives, cryptobeilic acids A–D and tsangibeilin B from the bark of *Endiandra cryptocaryoides* [20]. These compounds showed moderate antiplasmodial activity against the chloroquinone-resistant *Plasmodium falciparum* strain NF54, and antibacterial activities against *Escherichia coli*, *Acinetobacter calcoaceticus* and *Pseudomonas stutzeri* [20]. 

The chemical investigation of the methanolic extract of *E. kingiana* bark extract led to the isolation of seven new endiandric acids, kingianic acids A–G (**1**–**7**), together with endiandric acid M (**8**), tsangibeilin B (**9**) and endiandric acid (**10**) (compound **10** was only found in the “PubChem” database (CID 71521970) without an associated reference regarding its origin and its spectroscopic data, so this compound is fully described in the present manuscript). Herein, the isolation and structure elucidation of the new tetracyclic endiandric acids; kingianic acids A-G, and the cytotoxic activities, Bcl-xL and Mcl-1 affinities of compounds **1**, **3**, **5**–**9** are reported.

## 2. Results and Discussion

**Figure 1:**
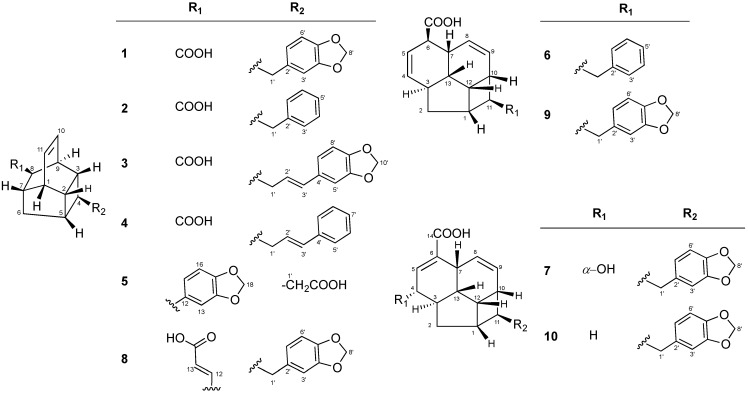
Structures of compounds **1**–**10**.

The EtOAc-soluble part of the *E. kingiana* methanol extract was subjected to silica gel chromatography to afford eight fractions Fr.1-Fr.8. Fractions Fr.4 and Fr.5 were further purified using silica gel as well as semi-preparative HPLC leading to the isolation of the kingianic acid series **1**–**7**, endiandric acid M (**8**), tsangibeilin B (**9**) and endiandric acid **10** (Figure 1). All assignments of ^1^H- and ^13^C-NMR data were then established through in depth analysis of 2D-NMR; NOESY, COSY, HSQC and HMBC experiments. All compounds **1**–**10** were isolated as optically inactive, thus suggesting that they are racemic mixtures and their spectroscopic data were very similar. They all possess a 13 carbon atom fused rings system and they can be divided into two main skeletal types. Six of compounds (**1**–**5**, **8**) belong to the first type as can be seen in endiandric acid K and endiandramide A [22]. While compounds **6**, **7**, **9** and **10** belong to the second type, similar to tsangibeilins A and B and endiandramide B [22]. 

Kingianic acid A (**1**) was isolated as a colorless oil. The HRESIMS spectrum of **1** showed a pseudomolecular ion peak [M−H]^−^ at *m/z* 323.1279 (calcd. 323.1284), consistent with a molecular formula C_20_H_20_O_4_ with 11 degrees of unsaturation. Its UV spectrum showed absorption bands at 233 and 286 nm, suggesting the presence of a benzenoid moiety, and its IR spectrum indicated the presence of OH (3,431 cm^−1^), carbonyl (1,701 cm^−1^) and methylenedioxy (1,040 and 936 cm^−1^) groups [22]. The ^13^C-NMR and DEPT spectra exhibited 20 signals, including 13 methines, three methylenes, and four quaternary carbons. The resonances of the methines at C-1 (*δ*_C_ 41.8), C-2 (*δ*_C_ 39.7), C-3 (*δ*_C_ 38.8), C-4 (*δ*_C_ 40.6), C-5 (*δ*_C_ 39.8), C-7 (*δ*_C_ 38.3), C-8 (*δ*_C_ 48.8) and C-9 (*δ*_C_ 34.8), including two olefinic carbons at C-10, C-11 and a methylene at C-6 observed in the DEPT spectrum, were characteristic of a tetracyclic endiandric acid like endiandric acid K [22]. The ^1^H and ^13^C-NMR spectra of **1** (Table 1) were also similar to those of endiandric acid K [22], except for the methylene proton signals [*δ*_H_ 2.72 (m, H-1'a); 2.78 (m, H-1'b)]. This group is placed between C-4 of the tetracylic acid moiety and C-2' of the benzenoid moiety in **1,** instead of three methylenes in endiandric acid K. 

**Table 1:** ^1^H (600 MHz) and ^13^C (150 MHz) NMR data of compounds **1**–**3** (CDCl_3_).

Position	1		2		3	
	δ_H_ (*J* in Hz)	δ_c_	δ_H_ (*J* in Hz)	δ_c_	δ_H_ (*J* in Hz)	δ_c_
1	2.71 m	41.8	2.72 dd (1.62, 5.1)	41.8	2.41 m	39.9
2	2.42 dt (8.5, 5.5)	39.7	2.44 dt (9.3, 3.7)	39.7	2.72 m	41.8
3	1.73 m	38.8	1.76 m	38.9	1.74 m	38.9
4	2.00 t (8.5)	40.6	2.07 t (8.0)	40.2	1.85 t (7.5)	39.0
5	2.34 t (6.5)	39.8	2.37 t (6.0)	39.9	2.38 m	39.8
6	1.55 d (13.0) 1.90 ddd (13.0, 7.5, 5.5)	38.5	1.54 d (12.8) 1.90 ddd (13.0, 7.4, 5.4)	38.4	1.58 d (12.8) 1.93 dt (7.1, 12.9)	38.4
7	2.57 t (5.0)	38.3	2.56 t (4.9)	38.3	2.57 t (5.5)	38.3
8	2.86 d (3.5)	48.8	2.87 d (2.6)	48.8	2.89 d (3.2)	48.6
9	2.98 dt (7.0, 4.0)	34.8	2.98 m	34.8	3.05 t (3.6)	34.8
10	6.22 t (4.0)	131.3	6.22 m	131.3	6.24 t (3.7)	131.9
11	6.22 t (4.0)	132.0	6.22 m	132.0	6.24 t (3.7)	132.3
1'	2.72 m 2.78 m	41.7	2.83 m 2.86 m	41.9	2.35 m	39.3
2'	-	134.7	-	140.9	6.02 dt (6.9, 15.7)	126.9
3'	6.66 s	109.1	7.15 d (7.1)	128.7	6.29 d (15.7)	130.5
4'	-	147.5	7.28 t (7.6)	128.3	-	131.4
5'	-	145.7	7.19 t (7.3)	125.8	6.90 d (1.2)	108.4
6'	6.72 d (8.0)	108.1	7.28 t (7.6)	128.4	-	148.0
7'	6.60 d (8.0)	121.5	7.15 d (7.1)	128.6	-	146.7
8'	5.92 s	100.8			6.73 d (8.0)	108.2
9'					6.76 d (8.0)	120.3
10'					5.94 s	101.0
C=O	-	179.3	-	179.4	-	178.3

**Figure 2:**
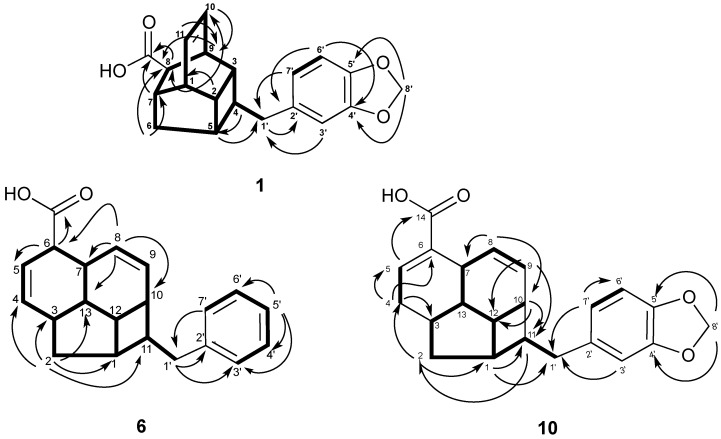
Key ^1^H-^1^HCOSY (bold) and HMBC (^1^H→^13^C) correlations of **1**, **6** and **10**.

**Figure 3:**
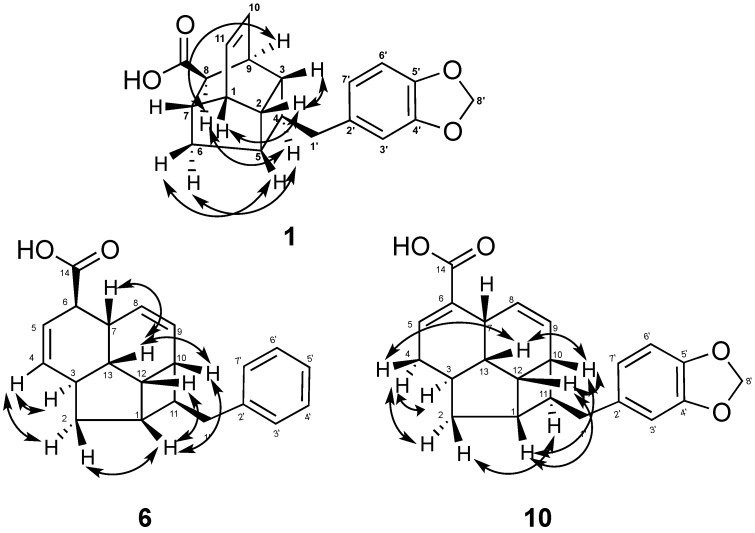
Selected NOESY (^1^H↔^1^H) correlations of **1**, **6** and **10**.

The ^1^H NMR of **1** revealed two *cis* olefinic proton signals at *δ*_H_ 6.22 (t, *J* = 4.0 Hz, H-10 and H-11). The three aromatic protons resonated as one singlet at *δ*_H_ 6.66 (s, H-3') and two *ortho*-coupled doublets *δ*_H_ 6.72 (d, *J* = 8.0 Hz, H-6') and 6.60 (d, *J* = 8.0 Hz, H-7') suggested the presence of a 1,3,4-trisubstituted aromatic ring. In addition, proton signals at *δ*_H_ 5.92 (s, H-8') confirmed the presence of the methylenedioxy group. As determined from the HMBC spectrum, the long-range correlations between H-7 (*δ*_H_ 2.57) and H-8 (*δ*_H_ 2.86) to COOH (*δ*_C_ 179.3) indicated the presence of carboxylic acid moiety attached to C-8 position of the tetracyclic moiety. Finally, the correlations of H-3'/C-1' (*δ*_C_ 41.7), H-7'/C-1', H-1'/C-2' (*δ*_C_ 134.7) and H-5/C-1' determined the connection of the methylenedioxyphenyl moiety to the tetracycle core through C-1' (Figure 2). The relative configuration of **1** was deduced from NOESY analysis (Figure 3) in combination with biogenetic consideration and comparison with endiandric acid K [22]. Based on NOESY spectrum, the *α*-orientation of H-9 was deduced from the correlations of H-9/H-8 and H-8/H-4. In contrast, other correlations between H-3/H-2, H-2/H-1 and H-5/H-6*β* and H-6 *β*/H-7 suggested that protons H-1, H-2, H-3, H-5 and H-7 to be *β*-oriented. Thus, the relative configuration was assigned as *rel-*(1*RS*, 2*RS*, 3*RS*, 4*SR*, 5*SR*, 7*SR*, 8*RS*, 9*SR*), the same as that of endiandric acid K [22]. 

Kingianic acid B (**2**) was obtained as a yellowish oil. The HRESIMS of **2** showed a pseudomolecular ion peak [M−H]^−^ at *m/z* 279.1398 (calcd. 279.1385) which indicated the molecular formula C_19_H_20_O_2_, and was consistent with 10 degrees of unsaturation. The UV, IR, ^1^H and ^13^C-NMR data (Table 1) were similar to those of **1**. The ^13^C-NMR and DEPT spectra exhibited 11 signals for an endiandric acid moiety, including the presence of 10 methines and one methylene group. The NMR data of compound **2** were nearly identical to those of **1**, except for the substituent at C-4, indicating that the methylenedioxyphenyl in **1** was replaced by a monosubstituted phenyl moiety in **2** [*δ*_H_ 7.15 (d, *J =* 7.1 Hz, H-3' and H-7'); 7.28 (t, *J =* 7.6 Hz, H-4' and H-6') and 7.19 (t, *J =* 7.3 Hz, H-5')]. Analysis of the NOESY spectrum indicated that **2** possess the same relative configuration as **1**.

Kingianic acids C (**3**) and D (**4**) were isolated as yellowish oils. The molecular formula of **3** (C_22_H_22_O_4_) and **4** (C_21_H_22_O_2_) were established by the [M−H]^−^ ion peaks at *m/z* 349.1439 (calcd. 349.1440) and 305.1539 (calcd. 305.1542) in the HRESIMS, respectively. The ^1^H and ^13^C-NMR data (Table 1 and Table 2) of compounds **3** and **4** were very similar to both **1** and **2**, and the analysis of the COSY correlations of **3** and **4** revealed the characteristic signals for a tetra-fused ring system as seen in compounds **1** and **2**. The presence of a carbonyl group was indicated by the absorption band at 1,697 cm^−1^ in the IR spectrum of **3** and 1,693 cm^−1^ for **4**, and confirmed by signals at *δ*_C_ 178.3 and 177.5 in their ^13^C-NMR spectra, respectively. In the HMBC spectra of **3** and **4**, the correlations from H-8 and H-7 to COOH confirmed the connectivity of the carboxylic acid function. Besides the characteristic tetracyclic moiety, analysis of COSY and HMBC correlations indicated that the methylenedioxyphenyl and monosubstituted phenyl moieties were attached at C-4 in compounds **3** and **4**, respectively. The geometry of C-2', 3' double bond was assigned as *trans* on the basis of the coupling constants of H-3' (*δ*_H_ 6.29, d, *J* = 15.7 Hz) and (*δ*_H_ 6.38, d, *J* = 15.8 Hz) in both compounds (Table 1 and Table 2). The NOESY spectra of **3** and **4**, showed correlation between H-8 and H-4 indicating that the benzyl moiety at C-4 assumed a *β*-position on the tetracyclic framework. Thus, the relative configuration of **3** and **4** were determined to be *rel-*(1*RS*, 2*RS*, 3*RS*, 4*SR*, 5*SR*, 7*SR*, 8*RS*, 9*SR*), the same as in compounds **1** and **2**. 

Kingianic acid E (**5**) was isolated as a yellowish oil. HRESIMS of **5** gave an [M−H]^-^ ion at *m/z* 323.1298 (calcd 323.1284), consistent with a molecular formula of C_20_H_20_O_4_ with 11 degrees of unsaturation. Its UV and IR spectra were similar to those of **1**. A close examination of the NMR spectra of compound **5** (Table 2) indicated that **5** possessed the same tetracyclic moiety as **1**, but with different substituents at C-4 and C-8. The two substituents consisted of an acetic acid group [*δ*_H_ 2.64 (*m*, H_2_-1'); *δ*_C_ 176.9 (COOH)] and a methylenedioxyphenyl moiety (*δ*_H_ 6.61, s, H-13; 6.67, d, *J* = 8.0 Hz, H-16; 6.54, d, *J* = 8.0 Hz, H-17; and 5.89, *s*, H-18). Their location at C-4 and C-8, respectively, was deduced from COSY and HMBC correlations. The COSY correlation between H-4 and H_2_-1' on one hand, and HMBC correlations from H-8 (*δ*_H_ 3.25) to C-12 (*δ*_C_ 140.2), and from H-13 (*δ*_H_ 6.61)/H-17 (*δ*_H_ 6.54) to C-8 (*δ*_C_ 47.9) on the other hand, confirmed the location of the acetic acid group and the methylenedioxyphenyl moiety at C-4 and C-8, respectively. The relative configuration of compound **5**, named kingianic acid E, was fixed by a NOESY experiment as the same as that of compound **1**.

The molecular formula C_21_H_22_O_2_ of kingianic acid F (**6**) was determined by HRESIMS analysis; 305.1557 (calcd 305.1542), for which 10 degrees of unsaturation could be deduced. The UV spectrum of **6** showed characteristic absorption bands at 232 and 288 nm suggesting the presence of a benzenoid moiety, and its IR spectrum showed absorption bands at 3,432 cm^−1^ for an OH group and 1,696 cm^−1^ for a carbonyl group. The ^13^C-NMR and DEPT spectrum, which showed 21 signals for two methylenes, 16 methines (nine olefinic), and two quaternary and a carbonyl carbon that were characteristic of the tetracyclic endiandric acid skeleton as seen in tsangibeilin B (**9**) [22]. The ^1^H and ^13^C-NMR spectra of compound **6** were reminiscent to those of **9**, except for the absence of signal for a methylenedioxy group in **6** and the appearance of five aromatic proton and carbon signals, thus suggesting that the methylenedioxyphenyl moiety was replaced by a monosubstituted phenyl moiety. All carbon-carbon connectivities of compound **6** were determined through a thorough analysis of 2D NMR spectra (Figure 2 and Figure 3), and comparison with those of tsangibeilin B (**9**). The location of the COOH group at C-6, and the phenyl moiety at C-11, was confirmed by HMBC correlation from H-6 to the carbonyl carbon at *δ*_C_ 179.4, and from COSY correlations between H-11 and H_2_-1', respectively. The relative configuration of **6** was ascertained by the careful inspection of NOESY spectrum (Figure 3) and biogenesis considerations of tsangibeilin B as reference [22]. 

**Table 2:** ^1^H (600 MHz) and ^13^C (150 MHz) NMR data of compounds **4** and **5** (CDCl_3_).

Position	4		5	
	δ_H_ (*J* in Hz)	δ_c_	δ_H_ (*J* in Hz)	δ_c_
1	2.73 m	41.9	2.76 dd (5.1, 10.8)	42.4
2	2.40 m	39.8	2.46 m	39.9
3	1.76 m	39.0	2.62 m	40.3
4	1.88 t (7.4)	38.9	2.44 m	35.3
5	2.36 t (7.4)	39.9	2.37 t (6.6)	40.2
6	1.61 d (12.7) 1.93 m	38.4	1.74 d (12.6) 1.94 m	39.3
7	2.58 t (5.1)	38.3	2.31 t (4.6)	43.1
8	2.90 d (3.8)	48.6	3.25 d (2.5)	47.9
9	3.06 t (3.8)	34.8	2.72 m	39.6
10	6.23 d (3.1)	131.4	5.93 t (7.0)	132.3
11	6.24 d (3.0)	131.9	6.29 t (7.3)	130.5
12				140.2
13			6.61 s	109.5
14				146.9
15				145.2
16			6.67 d (8.0)	107.4
17			6.54 d (8.0)	121.6
18			5.89 s	100.7
1'	2.43 m	39.4	2.64 m	40.4
2'	6.19 m	128.7		
3'	6.38 d (15.8)	131.0		
4'	-	137.7		
5'	7.35 d (7.2)	126.0		
6'	7.30 t (7.6)	128.5		
7'	7.20 t (7.3)	127.0		
8'	7.30 t (7.6)	128.5		
9'	7.35 d (7.2)	126.0		
10'				
C=O	-	177.5		176.9

Thus, the relative configuration of **6**, named kingianic acid F or 11{1'-[phenyl]}-tetracyclo[5.4.2.0^3,13^.0^10,12^]trideca-4,8-dien-6-carboxylic acid (kingianic acid F), was assigned as *rel-*(1*RS*, 2*RS*, 3*RS*, 4*SR*, 5*SR*, 7*SR*, 8*RS*, 9*SR*), same as that of tsangibeilin B (**9**) [22].

Kingianic acid G (**7**) was obtained as a yellowish oil. The negative-mode HREIMS exhibited a quasi-molecular ion peak at *m/z* 365.1401 [M−H]^−^ (calcd. 365.1389), which suggested the molecular formula of C_22_H_22_O_5_, and implied 12 degrees of unsaturation. The UV absorption bands at λ_max_ 234 and 286 nm confirmed the presence of a benzenoid nucleus [23]. The absorption bands at 2,600–3,300, 1,687, 1,632, 1,040 and 937 cm^−1^ in the IR spectrum revealed the presence of OH, C=O, C=C and O-CH_2_-O groups, respectively. The ^1^H and ^13^C-NMR data of **7** were similar to those of **10**, suggesting that they both share a common tetracyclic endiandric acid skeleton, except for the presence of an α-OH at δ_H_ 4.23 (d, *J* = 9.8 Hz, H-4) in **7**. The location of this hydroxy group at C-4 was deduced from the COSY correlations between H-3/H-5 and H-4, and HMBC correlations from H-2a (δ_H_ 1.29), H-3 and H-13 to C-4. The relative configuration of the stereogenic centers was established by biogenetic considerations [24], analysis of the NOESY spectrum and comparison with NMR data reported in the literature [23]. NOESY correlations between H-12 and H-1/H-10/H-13, H-1, and between H-7 and H-8/H-13 confirmed that protons H-1, H-7, H-10, H-12 and H-13 are all cofacial, arbitrarily assigned as *β*-oriented. Since cross peaks between H-4 at δ_H_ 4.23 and H-2a and H-2b, at δ_H_ 1.29 and 1.79 respectively, were equally intense, the relative configuration at C-4 could not be deduced from the NOESY spectrum. However, comparison of NMR data of kingianic acid G (**7**) with data reported in the literature for beilschmiedic acids H and I allowed the assignment of the 4-OH configuration [23]. Indeed, since compound **7** possesses similar ^13^C chemical shifts to beilschmiedic acid H for carbons C-3, C-4 and C-5 (δ_C_ 44.1, 73.6, 145.6, respectively for **7**; δ_C_ 45.1, 74.5 and 145.8, respectively for beilschmiedic acid H), it can be deduced that the hydroxy group is *α-*oriented, as was the case for beilschmiedic acid H [23]. In the case of beilschmiedic acid I, having a *β*-oriented hydroxy group at C-4, chemical shifts of C-3, C-4 and C-5 are more shielded; 43.1, 65.9 and 141.5 ppm, respectively [23]. Thus, kingianic acid G (**7**) was proposed to have the same relative configuration as beilschmiedic acid H.

Compound **10** was obtained as an amorphous solid. The HRESIMS spectrum of **10** showed a pseudomolecular ion peak [M−H]^−^ at *m/z* 349.1431(calcd. 349.1440), consistent with a molecular formula of C_22_H_22_O_4_, with 12 degrees of unsaturation. Its IR spectrum showed strong absorption bands at 1,685 cm^−1^ for C=O, and 1,630 cm^−1^ for C=C, and UV absorption bands at λ_max_ 234 and 286 nm suggesting the presence of a benzenoid moiety. The ^13^C-NMR spectrum of **10** (Table 3) contained 22 carbons signals, which were sorted by DEPT-135 NMR and HSQC into 5 quaternary carbons, 14 methines and 4 methylene groups. Resonances of methines at *δ*_C_ 40.9 (C-1), 35.6 (C-3), 144.6 (C-5), 33.3 (C-7), 127.1 (C-8), 127.0 (C-9), 33.2 (C-10), 46.9 (C-11), 34.0 (C-12) and 42.2 (C-13), and methylene groups at *δ*_C_ 37.0 (C-2) and 32.2 (C-4) observed in the DEPT spectrum, were characteristic of a tetracyclic endiandric acid skeleton as seen in cryptobeilic acids A-D [20]. The ^13^C-NMR spectrum showed also signals of a conjugated carbonyl group at *δ*_C_ 178.0 (COOH), an olefinic quaternary carbon at *δ*_C_ 134.6 (C-6) and carbons of a substituted methylenedioxybenzyl moiety (C-1'-C-8'). The presence of olefinic methines in **10** was confirmed by the ^1^H-NMR spectrum (Table 3), which showed a broad singlet of proton H-5 at *δ*_H_ 7.23 (brs, H-5) and a pair of protons appearing as doublet at *δ*_H_ 5.39 (d, *J* = 10.0 Hz, H-8) and 5.54 (d, *J* = 10.0 Hz, H-9). The *ortho* coupled proton H-6' and H-7' resonated as a pair of doublets at *δ*_H_ 6.71 (d, *J* = 7.9 Hz, H-6') and 6.60 (d, *J* = 7.9 Hz, H-7'), while H-3' appeared as a singlet at *δ*_H_ 6.65 (s, H-3'). In addition, protons signal at *δ*_H_ 5.91 (s, H-8') confirmed the presence of the methylenedioxy group. HMBC correlations (Figure 2) from H-5 to C-6 (*δ*_C_ 134.6) and COOH (*δ*_C_ 178.0), and from H-8 to C-6 established the location of the carboxylic group at C-6. The attachment of the methylenedioxybenzyl moiety at H-11 was confirmed by the correlation from H-11 to C-2', and from H-1' to C-10 and C-1. The relative configuration of the asymmetric carbons was established by NOESY analysis (Figure 3) and confirmed by X-ray crystallographic analysis (see Supporting Information). Therefore, the relative configuration of H-1, H-3, H-6, H-7, H-10, H-11, H-12, and H-13 was assigned as *rel*-(1*SR*,3*RS*,7*RS*,10*RS*,11*SR*,12*SR*,13*SR*), same as that of beilschmiedic acid H [23]. 

**Table 3:** ^1^H (600 MHz) and ^13^C (150 MHz) NMR data of compounds **6**, **7**, and **10** (CDCl_3_).

Position	6		7		10	
	δ_H_ (*J* in Hz)	δ_c_	δ_H_ (*J* in Hz)	δ_c_	δ_H_ (*J* in Hz)	δ_c_
1	2.45 m	41.0	2.48 m	40.9	2.42 m	40.9
2	1.30 dt (6.3, 12.1) 1.53 dd (6.4, 11.9)	34.7	1.29 m 1.79 m	35.0	1.20 m 1.53 m	37.0
3	2.55 m	36.9	2.08 m	44.1	2.04 m	35.6
4	6.19 d (9.7)	134.4	4.23 d (9.8)	73.6	2.08 m, 2.47 m	32.2
5	5.72 d (9.6)	123.9	7.03 brs	145.6	7.23 brs	144.6
6	3.00 m	49.0	-	134.3	-	134.6
7	2.84 m	32.8	3.23 brs	33.2	3.26 brs	33.3
8	5.40 m	129.8	5.43 d (10.5)	127.7	5.39 d (10.0)	127.1
9	5.42 m	129.1	5.53 d (10.5)	126.2	5.54 d (10.0)	127.0
10	2.39 m	34.5	2.42 m	33.7	2.40 m	33.2
11	1.82 m	46.9	1.76 m	46.8	1.74 m	46.9
12	2.70 dd (7.7, 16.2)	32.9	2.80 m	33.6	2.77 m	34.0
13	1.73 m	42.0	1.86 m	40.8	1.68 m	42.2
1'	2.81 m	42.9	2.71 d (8.0)	42.5	2.70 d (8.0)	42.6
2'	-	140.7	-	134.6	-	134.6
3'	7.15 d (6.8)	128.6	6.64 s	108.9	6.65 s	109.0
4'	7.24 t (6.7)	128.4	-	147.5	-	147.4
5'	6.20 dt (2.1, 7.3)	125.8	-	145.6	-	145.6
6'	7.24 t (6.7)	128.4	6.71 d (7.9)	108.1	6.71 d (7.9)	108.0
7'	7.15 d (6.8)	128.6	6.59 d (7.9)	121.3	6.60 d (7.9)	121.3
8'			5.91 s	100.7	5.91 s	100.7
C=O		179.4		170.3		178.0

Endiandric acid M (**8**) and tsangibeilin B (**9**) were readily identified by comparison with literature data [22,25]. The structure and relative configuration of tsangibeilin B (**9**) were confirmed by single-crystal X-ray analysis (see Supporting Information).

Compounds **1**, **3**, **5**–**9** were screened against the anti-apoptotic proteins Bcl-xL and Mcl-1 using fluorescence polarization assays according to Qian and co-workers [26]. Assays are based on the interaction of fluorescein-labeled peptides [the BH3 domain of BAK protein (F-Bak) or BID protein (F-Bid) to Bcl-xL and Mcl-1, respectively]. No binding was detected for Bcl-xL and only weak binding affinity for Mcl-1 (25%–30% inhibition at 20 µM and ≥ 75% at 100 µM) were obtained with compounds **3**, **6** and **9** (Table 4). In these assays, amount of compounds **2**, **4** and **10** were not sufficient for this biological evaluation.

**Table 4:** Biological activities of compounds **1**, **3**, **5**–**9**.

Compounds	Bcl-xL/Bak binding affinity (%)		Mcl-1/Bid binding affinity (%)		Cytotoxicity (IC_50_ in µM,mean ± s.d., *n* = 3)		
	20 μM	100 μM	20 μM	100 μM	HT-29	A549	PC3
**1**	3 ± 1.5	21 ± 1.8	3 ± 2.0	36 ± 2.3	35.0 ± 0.2	85.4 ± 0.2	>100
**3**	9 ± 1.5	25 ± 1.7	30 ± 2.2	75 ± 1.1	>100	85.3 ± 0.2	>100
**5**	2 ± 1.4	1 ± 0.8	3 ± 1.3	8 ± 5.5	17.1 ± 0.1	15.4 ± 0.2	77.2 ± 0.2
**6**	4 ± 1.6	22 ± 2.9	28 ± 3.7	80 ± 0.7	NT	NT	NT
**7**	5 ± 1.3	19 ± 1.6	8 ± 1.1	47 ± 2.9	NT	NT	NT
**8**	0	10 ± 0.5	4 ± 0.8	39 ± 0.9	>100	>100	>100
**9**	6 ± 1.5	26 ± 2.5	25 ± 2.1	81 ± 2.4	>100	38.1 ± 0.1	>100
**U-Bak (Ki)**		12 ± 1 nM					
**U-Bid (Ki)**				16 ± 2 nM			
**ABT-737 (Ki)**		57 ± 10 nM		47 ± 22 nM			
**Cisplatin**					70.3 ± 1.1	36.2 ± 1.4	44.5 ± 7.7

NT: not tested; U-Bak and U-bid correspond to unlabeled peptides Bak and Bid, respectively.

Compounds **1**, **3**, **5**, **8** and **9** were screened for cytotoxic activity against A549 (lung adenocarcinoma epithelial), HT29 (colorectal adenocarcinoma) and PC3 (prostate adenocarcinoma) cell lines using a 3-(4,5-dimethylthiazol-2-yl)-5-(3-carboxymethoxyphenyl)-2-(4-sulfophenyl)-2*H*-tetrazolium, inner salt (MTS)-based assay (Table 4). Compound **5** showed moderate cytotoxic activity against lung adenocarcinoma epithelial (A549) and colorectal adenocarcinoma cell lines (HT-29) with IC_50_ of 15.36 ± 0.19 µM and 17.10 ± 0.11 µM, respectively. The other compounds showed very weak or devoid of (A549) cytotoxic activity against the cancer cell lines tested. Our results are in agreement with a previous study by Williams, in which some synthetic tetracyclic endiandric acids were not active on prostate adenocarcinoma cancer cells (PC3), but significantly active on lung carcinoma cells [23].

## 3. Experimental

### 3.1. General

Optical rotations were measured on a JASCO P-1020 polarimeter. IR spectra (neat) were taken on a Perkin Elmer RX1 FT-IR spectrometer. 1D (^1^H, ^13^C, DEPT) and 2D (COSY, NOESY, HSQC, HMBC) NMR experiments were carried out on a Bruker Avance 600 (600 MHz for ^1^H NMR, 150 MHz for ^13^C NMR) spectrometer. Data were analysed via TopSpin software package. Chemical shifts were internally referenced to the solvent signals in CDCl_3_ (^1^H, δ 7.26; ^13^C, δ 77.0). High-resolution ESIMS on a Thermoquest TLM LCQ Deca ion-trap mass spectrometer. Silica gels (230–400 mesh) (Merck) were used for column chromatography (CC), and silica gel 60 F-254 (Merck) was used for analytical TLC. Agilent^®^ Eclipse Zorbax C18 column (250 × 9.4 mm, 3.5 µm) and Waters^®^ X-Bridge C18 column (250 × 10.0 mm, 5.0 µm); were used for semi-preparative HPLC separations using a Waters auto purification system equipped with a sample manager (Waters 2767), a column fluidics organizer, a binary pump (Waters 2525), a UV–Vis diode array detector (190–600 nm, Waters 2996), and a PL-ELS 1000 ELSD Polymer Laboratory detector. X-ray data collection was obtained from a BrukerAPEX2; cell refinement: SMART [27]; data reduction: SAINT [27]; program(s) used to solve the structure: SHELXTL [28]; program(s) used to refine structure: SHELXTL [28].

### 3.2. Plant Material

The bark of *Endiandra kingiana* Gamble was collected at Reserved Forest Sg. Temau, Kuala Lipis, Pahang, Malaysia in May 2006. This plant was identified by T. Leong Eng Botanist University of Malaya. A voucher specimen (KL-5243) has been deposited at the Herbarium of the Department of Chemistry, Faculty of Science, University of Malaya, Kuala Lumpur, Malaysia.

### 3.3. Extraction and Isolation

The air-dried bark of E. kingiana (1.5 kg) were sliced, ground and extracted with EtOAc (3 × 1.5 L) followed by MeOH (3 × 1.5 L) at 40 °C and 100 bar using a Zippertex static high-pressure, high-temperature extractor developed at the ICSN pilot unit. The methanol extract was concentrated under reduced pressure and was partitioned with EtOAc/H_2_O (1:1, v/v) to afford an EtOAc-soluble fraction (22.5 g) and a H_2_O-soluble fraction (91.7 g). The EtOAc-soluble fraction (22 g) was subjected to column chromatography (CC, 660 g SiO_2_, 230–400 mesh; hexane/dichloromethane/methanol step gradient) to give eight fractions, identified as Fr. 1–Fr. 8.

Fr. 4 (4.3 g) was subjected to CC (129 g, SiO_2_, 230–400 mesh; hexane/AcOEt step gradient) to obtain 20 subfractions base on TLC profile: Fr. 4.1–Fr. 4.20. Fraction Fr. 4.3 (37.8 mg) was separated using semi-preparative C_18_ HPLC eluted at 3.5 mL/min isocratically with MeCN-H_2_O 65:35 + 0.1% formic acid from 5 to 50 min (Agilent^®^ Eclipse Zorbax C_18_ column (250 × 9.4 mm, 3.5 µm). Serial collections afforded **2** (*t_R_* 8.0 min, 1.2 mg) and **4** (*t_R_* 16.0 min, 1.0 mg). **1** (*t_R_* 13.1 min, 12.1 mg), **6** (*t_R_* 24.8 min, 3.4 mg) and **10** (*t_R_* 19.9 min, 1.2 mg) were purified from the fraction Fr. 4.4 (71.0 mg) with a semi-preparative C-18 column (Agilent^®^ Eclipse Zorbax C18 column (250 × 9.4 mm, 3.5 µm) using MeCN-H_2_O 65:35 plus 0.1% formic acid at 3.5 mL/min. Further analogues were separated from fraction Fr. 4.7 (41.4 mg) and separated using semi-preparative C-18 column (Agilent^®^ Eclipse Zorbax C18 column (250 × 9.4 mm, 3.5 µm) using MeCN-H_2_O 65:35 plus 0.1% formic acid at 3.5 mL/min. Serial collections afforded **9** (*t_R_* 14.7 min, 1.8 mg) and **3** (*t_R_* 16.4 min, 4.4 mg). From fraction Fr 4.14 (72.4 mg), **5** (*t_R_* 14.6 min, 1.4 mg) and **8** (*t_R_* 25.1 min, 2.0 mg) were isolated using semi-preparative HPLC eluted at 3.5 mL/min isocratically with MeCN-H_2_O 60:40 plus 0.1% formic acid (Agilent^®^ Eclipse Zorbax C18 column (250 × 9.4 mm, 3.5 µm). Fr. 5 (0.7 g) was subjected to CC (21 g, SiO_2_, 230–400 mesh; hexane/AcOEt step gradient) to obtain 12 subfractions according to their TLC profiles. Fraction Fr. 5.7 (26.8 mg) was purified using a semi-preparative C_18_ column (Waters^®^ X-Bridge C18 column (250 × 10.0 mm, 5.0 µm) using MeCN-H_2_O 50:50 plus 0.1% formic acid at 3.0 mL/min afforded **7** (*t_R_* 13.4 min, 1.5 mg). 

### 3.4. Spectral Data

*Kingianic acid* A (**1**): yellowish oil; 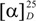 ± 0 (c 0.20, CHCl_3_); UV (MeOH) λ_max_ 233, 286 nm; IR (neat) ν_max_ 3431(OH), 1701 (C=O), 1040, 936 (OCH_2_O) cm^−1^; ^1^H-NMR and ^13^C-NMR, see Table 1; HRESIMS *m*/*z* 323.1279 [M−H]^−^ (calcd for C_20_H_19_O_4_, 323.1284).

*Kingianic acid B* (**2**): yellowish oil; 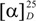 ± 0 (c 0.12, CHCl_3_); UV (MeOH) λ_max_ 212, 287 nm; IR (neat) ν_max_ 3432(OH), 1721 (C=O) cm^−1^; ^1^H-NMR and ^13^C-NMR, see Table 1; HRESIMS *m/z* 279.1398 [M−H]^−^ (calcd for C_19_H_19_O_2_, 279.1385).

*Kingianic acid C* (**3**): yellowish oil; 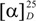 ± 0 (c 0.20, CHCl_3_); UV (MeOH) λ_max_ 212, 290 nm; IR (neat) ν_max_ 3437 (OH), 1697 (C=O), 1037, 923 (OCH_2_O) cm^−1^; ^1^H-NMR and ^13^C-NMR, see Table 1; HRESIMS *m/z* 349.1439 [M−H]^−^ (calcd for C_22_H_21_O_4_, 349.1440).

*Kingianic acid D* (**4**): yellowish oil; 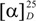 ± 0 (c 0.10, CHCl_3_); UV (MeOH) λ_max_ 212, 289 nm; IR (neat) ν_max_ 3440(OH), 1693 (C=O) cm^−1^; ^1^H-NMR and ^13^C-NMR, see Table 2; HRESIMS *m/z* 305.1539 [M−H]^−^ (calcd for C_21_H_21_O_2_, 305.1542).

*Kingianic acid E* (**5**): yellowish oil; 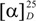 ± 0 (c 0.14, CHCl_3_); UV (MeOH) λ_max_ 234, 286 nm; IR (neat) ν_max_ 3444(OH), 1665 (C=O), 1039, 938 (OCH_2_O) cm^−1^; ^1^H-NMR and ^13^C-NMR, see Table 2; HRESIMS *m/z* 323.1298 [M−H]^−^ (calcd for C_20_H_19_O_4_, 323.1284).

*Kingianic acid F* (**6**): yellowish oil; 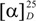 ± 0 (c 0.16, CHCl_3_); UV (MeOH) λ_max_ 232, 288 nm; IR (neat) ν_max_ 3432 (OH), 1696 (C=O) cm^−1^; ^1^H-NMR and ^13^C-NMR, see Table 3; HRESIMS *m/z* 305.1557 [M−H]^−^ (calcd for C_21_H_21_O_2_, 305.1542).

*Kingianic acid G* (**7**). Yellowish oil. 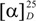 ± 0 (c 0.14, CHCl_3_). UV (MeOH) λ_max_ 234, 286 nm. IR (neat) ν_max_ 2600–3300 (OH), 1687 (C=O), 1632 (C=C) and 1040, 937 (OCH_2_O) cm^−1^. ^1^H-NMR and ^13^C-NMR, see Table 3. HREIMS: *m/z* 365.1401 [M−H]^−^ (calcd for C_22_H_21_O_5_, 365.1389). 

Compound **10**: Amorphous solid. 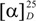 ± 0 (c 0.12, CHCl_3_). UV (MeOH) λ_max_ 234, 286 nm. IR (neat) ν_max_ 1685 (C=O), 1630 (C=C) and 1039, 935 (OCH_2_O) cm^−1^. ^1^H-NMR and ^13^C-NMR, see Table 3. HREIMS: *m/z* 349.1431[M−H]^−^ (calcd for C_22_H_21_O_4_, 349.1440). A colourless crystal was obtained from MeOH, crystallized in the monoclinic crystal system with P21/*c* space group. Cell parameters: a = 6.141(2)Å; b = 23.448(8) Å; c = 12.366(4) Å; *β* = 104.38˚; V = 1834.58(8) Å3, *T* 100 K. For the X-ray crystallographic data of compound **10** see Supporting Information. Supplementary crystallographic data have been deposited with the CCDC as CCDC-918161. 

CCDC-918161 contains the supplementary crystallographic data for this paper. These data can be obtained free of charge via http://www.ccdc.cam.ac.uk/conts/retrieving.html (or from the CCDC, 12 Union Road, Cambridge CB2 1EZ, UK; Fax: +44 1223 336033; E-mail: deposit@ccdc.cam.ac.uk).

### 3.5. Bcl-xL and Mcl-1 Binding Affinity Assays

The binding affinities of compounds for Bcl-xL and Mcl-1 were evaluated by competition against fluorescently labelled reference compounds, Bak and Bid, respectively, as described by Qian *et al.* [26]. Human 45-84/ C37 Bcl-xL and mouse DN150/DC25 Mcl-1 proteins were recombinantly produced by N. Birlirakis at ICSN. Bak, 5-Carboxyfluorescein-Bak, Bid and 5-carboxyfluorescein-Bid peptides were synthetized by PolyPeptide Laboratories (Strasbourg, France). All sequences are available in the Supporting Information (S37). Unlabeled peptides were dissolved in DMSO (Carlo Erba, Val de Reuil, France) and labelled peptides were diluted in assay buffer, which contained 20 mM Na_2_HPO_4_ (pH 7.4), 50 mM NaCl, 2 µM EDTA, 0.05% Pluronic F-68, without pluronic acid for storage at −20 °C. Liquid handling instrument, Biomek^®^NX and Biomeck^®^3000 (Beckman Coulter, Villepinte, France), were used to add protein and fluorescein-labelled peptides. 15 nM labelled BH3 peptide, 100 nM protein, and 100 μM of unlabelled BH3 peptide or compound (first diluted in 10 mM DMSO and then buffer for final concentration from 10^−9^ to 10^−4^ M) into a final volume of 40 µL were distributed in a 96 well black polystyrene flat-bottomed microplate (VWR 734-1622). The microplate was then incubated at room temperature for 1 h and shaken before fluorescent polarization measure. Fluorescence polarization in millipolarization units was measured with a Beckman Coulter Paradigm^®^ using FP cartridge (λ_ex_ 485 nm, λ_em_ 535nm). The exposure time was 300 ms per channel. All experimental data were collected using the Biomek Software^®^ (Beckman Coulter, Inc, Brea, CA, USA) and analysed using Microsoft Excel 2010 (Microsoft, Redmond, WA, USA). Results are expressed as binding activity, *i.e.*, percentage of inhibition of the binding of labelled reference compound, or as *Ki*, the concentration corresponding to 50% of such inhibition, and corrected for experimental conditions according to Kenakin rearranged equation [29], which is adapted from Cheng and Prusoff equation [30]. ABT-737, which was kindly provided by O. Nosjean (Institut de Recherche Servier, Croissy, France), and unlabeled peptides Bak and Bid were used as positives control. The performance of the assays was monitored by use of Z' factors as described by Zhang *et al.* [31]. The Z' factors for these assays are 0.8 (Bcl-xL/Bak) and 0.7 (Mcl-1/Bid) indicating that they should be robust assays. 

### 3.6. Cell Viability Assay

Human cancer cell lines A549 (Lung adenocarcinoma epithelial), HT29 (Colorectal adenocarcinoma) and PC3 (Prostate adenocarcinoma) cells were obtained from the ATCC (Manassas, VA, USA). Cells were grown in RPMI-1640 or DMEM medium with 10% FBS supplemented with 4 mM L-glutamine and 1% penicillin-streptomycin. For experimental purposes, the cells growing exponentially and maintained at 70%–80% confluency were used. Cells were seeded into 96-well plates at 10^4^ cells/well and allowed to adhere overnight; the medium was then removed. A stock solution of test compound in DMSO was diluted in medium to generate a series of working solutions. Aliquots (100 μL) of the working solutions were added to the appropriate test wells to expose cells to the final concentrations of compound in a total volume of 100 μL. Nine different concentrations (100 µL–0.4 µL) were tested, in triplicates. Cisplatin is used as a positive control and wells containing vehicle without compound were used as negative controls. Plates were kept for 48 h in a 37 °C, 5% CO_2_ incubator. After incubation, viable cells were detected with the CellTiter 96 AQueous cell proliferation assay (Promega Corp., Madison, WI, USA). Plates were read in a microplate reader (Tecan Infinite^®^ 200 PRO series, Mannedorf, Switzerland) at 490nm. Then, dose-response curves were generated and the IC_50_ values were determined using GraphPad Prism 5.04 software (La Jolla, CA, USA [32].

## 4. Conclusions

The phytochemical investigation of *Endiandra kingiana* methanolic bark extract has led to the isolation of seven new tetracyclic endiandric acid analogues, named kingianic acids A–G (**1**–**7**), together with endiandric acid M (**8**), tsangibeilin B (**9**) and compound **10**. These compounds were screened for Bcl-xL and Mcl-1 binding affinities, and cytotoxic activity on various cancer cell lines. Compound **5** showed moderate cytotoxic activity, with IC_50_ values in the range 15–17 µM, and compounds **3**, **6** and **9** exhibited weak binding affinity for the anti-apoptotic protein Mcl-1. This is the first report of binding affinity toward Mcl-1 for endiandric acid analogues.

## Supplementary Materials

Supplementary materials: HRESIMS and NMR spectra for compounds **1**–**7** and **10**, and the X-ray crystallographic analysis data of tsangibeilin B (**9**) and endiandric acid **10** can be accessed at: http://www.mdpi.com/1420-3049/19/2/1732/s1.
