# Supplementary material for: Kingianic Acids A–G, Endiandric Acid Analogues from Endiandra kingiana
Source: Molecules. 2014 Jan 31;19(2):1732–47. doi: 10.3390/molecules19021732 (PMC6270856; doi:10.3390/molecules19021732)
Supplement: Supplementary file 1 [file molecules-19-01732-s001.pdf]

Supplementary Materials

Supplementary Information.

Structures of kingianic acid A-G (**1**–**7**), endiandric acid M (8), tsangibeilin B (**9**), and endiandric acid **10**.	S2
X-ray crystallographic analysis of endiandric acid (**10**)	S3
X-ray crystallographic analysis of tsangibeilin B (**9**)	S4
HRESIMS for kingianic acid A (**1**)	S5
NMR spectra for kingianic acid A (**1**) in $CDCl_3$	S5–S6
HRESIMS for kingianic acid B (**2**)	S7
NMR spectra for kingianic acid B (**2**) in $CDCl_3$	S7–S8
HRESIMS for kingianic acid C (**3**)	S9
NMR spectra for kingianic acid C (**3**) in $CDCl_3$	S9–S10
HRESIMS for kingianic acid D (**4**)	S11
NMR spectra for kingianic acid D (**4**) in $CDCl_3$	S11–S12
HRESIMS for kingianic acid E (**5**)	S13
NMR spectra for kingianic acid E (**5**) in $CDCl_3$	S13–S14
HRESIMS for kingianic acid F (**6**)	S15
NMR spectra for kingianic acid F (**6**) in $CDCl_3$	S15–S16
HRESIMS for kingianic acid G (**7**)	S17
NMR spectra for kingianic acid G (**7**) in $CDCl_3$	S17–S18
HRESIMS for endiandric acid M (**8**)	S19
NMR spectra for endiandric acid M (**8**) in $CDCl_3$	S19–S20
HRESIMS for endiandric acid **10**	S21
NMR spectra for endiandric acid **10** in $CDCl_3$	S21–S22
Sequences alignment of Bcl-xL and Mcl-1 with Bak-BH3 and Bid-BH3 peptides respectively	S23

Figure S1. Structures of kingianic acids A-G (**1**–**7**), endiandric acid M (**8**), tsangibeilin B (**9**) and endiandric acid **10**.

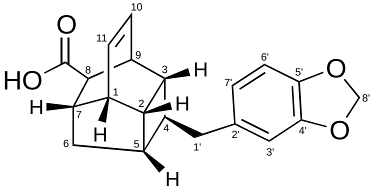

Kingianic acid A **(1)**

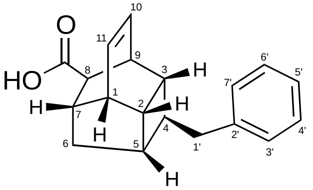

Kingianic acid B **(2)**

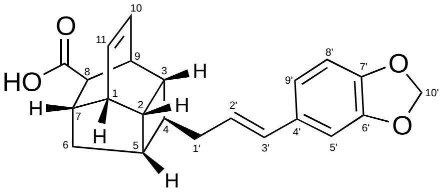

Kingianic acid C **(3)**

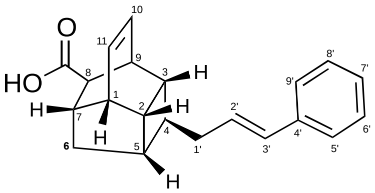

Kingianic acid D **(4)**

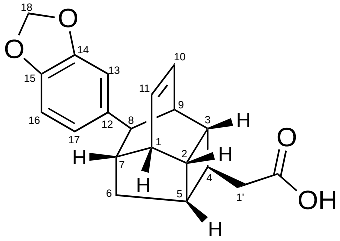

Kingianic acid E **(5)**

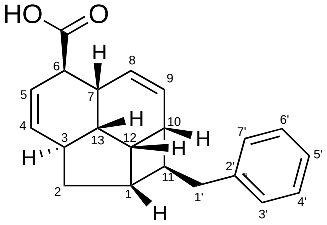

Kingianic acid F **(6)**

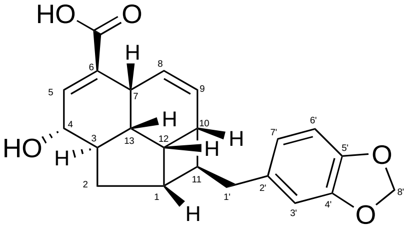

Kingianic acid G **(7)**

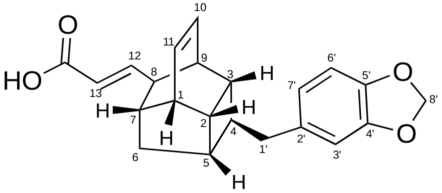

Endiandric acid **(8)**

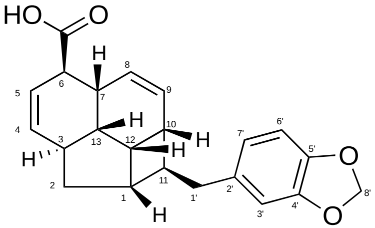

Tsangibelin B **(9)**

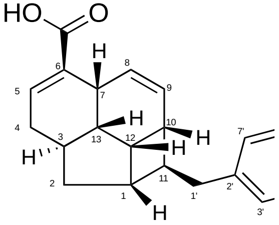

Endiandric acid **10**

Figure S2. X-ray crystallographic analysis of endiandric acid **10**: A colourless crystal was obtained from MeOH, crystallized in the monoclinic crystal system with P21/c space group. Cell parameters: a = 7.9900(2) Å; b = 21.9056(6) Å; c = 10.8257(3) Å; V = 1834.58(8) Å3, *T* 100 K. In the crystal structure, two molecules are linked by hydrogen bonds across a centre of inversion (**A**) and adjacent pairs are further linked by the double CH-π interactions that occur between the phenyl and the dioxole ring (**B**). The overall crystal packing gives rise to a 2-D layered structure as shown in **C**. Supplementary crystallographic data have been deposited at the CCDC; CCDC 918161. ORTEP plot of endiandric acid **10** with the displacement ellipsoids drawn at the 50% probability level.

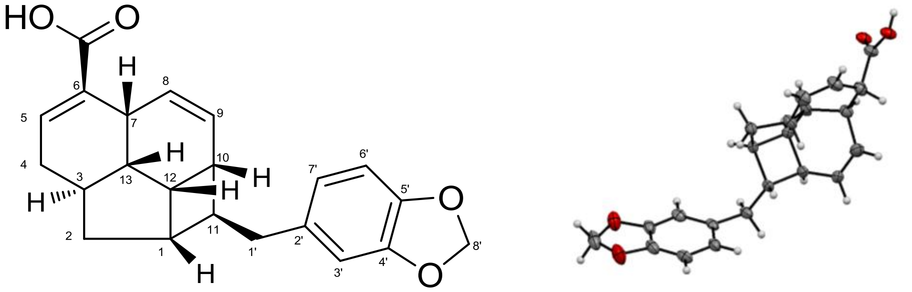

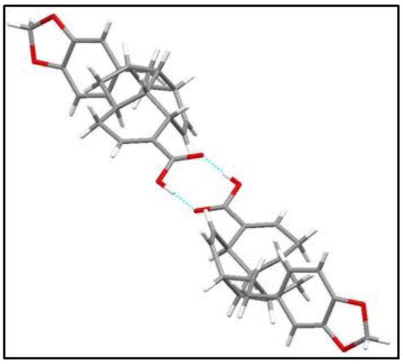

(**A**)

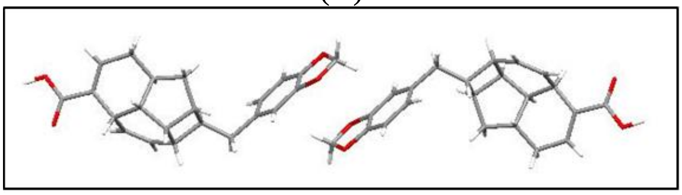

(**B**)

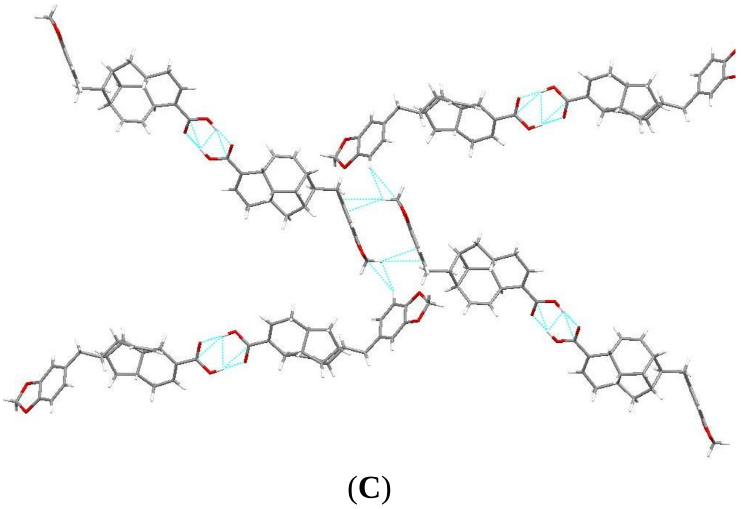

(**C**)

Figure S3. *X-Ray Crystallographic Data of tsangibeilin B (***9***)*. A colorless crystal was obtained from MeOH, crystallized in the monoclinic crystal system with C2/c space group. Cell parameters: a = 41.694(9) Å; b = 6.8688(15 Å; c = 12.282(3) Å; *β* = 101.742(4) °; V = 1834.58(8) Å3, , *T* 100 K.

ORTEP plot of tsangibeilin B (**9**) with the displacement ellipsoids drawn at the 50% probability level.

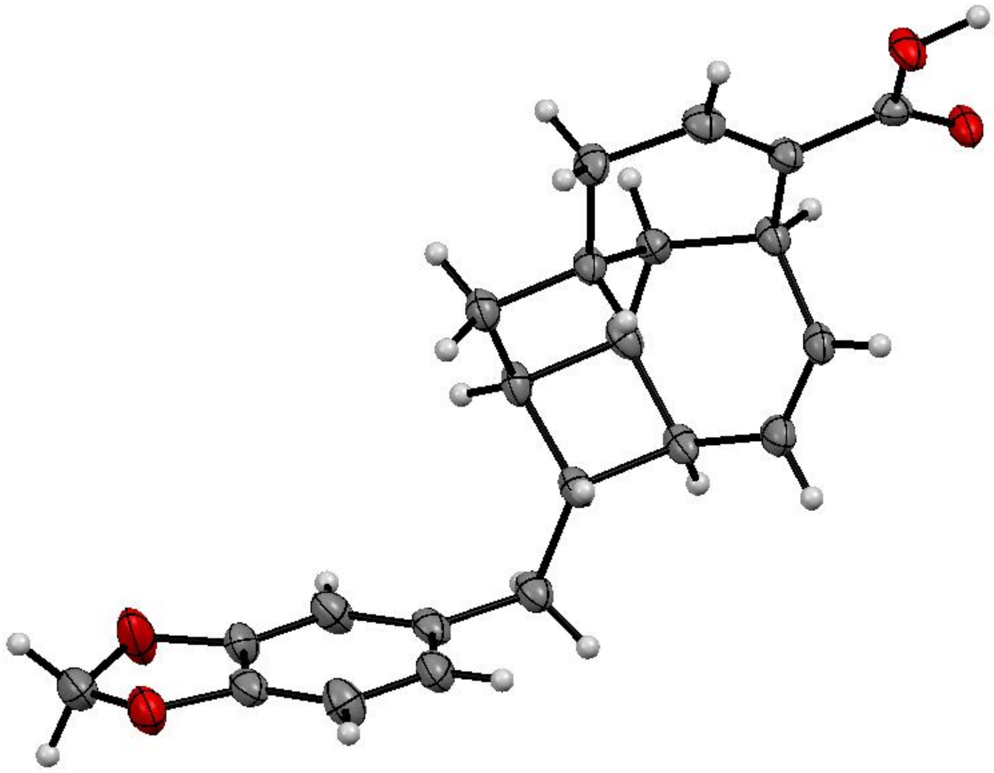

Figure S4. HRESIMS of kingianic acid A (**1**).

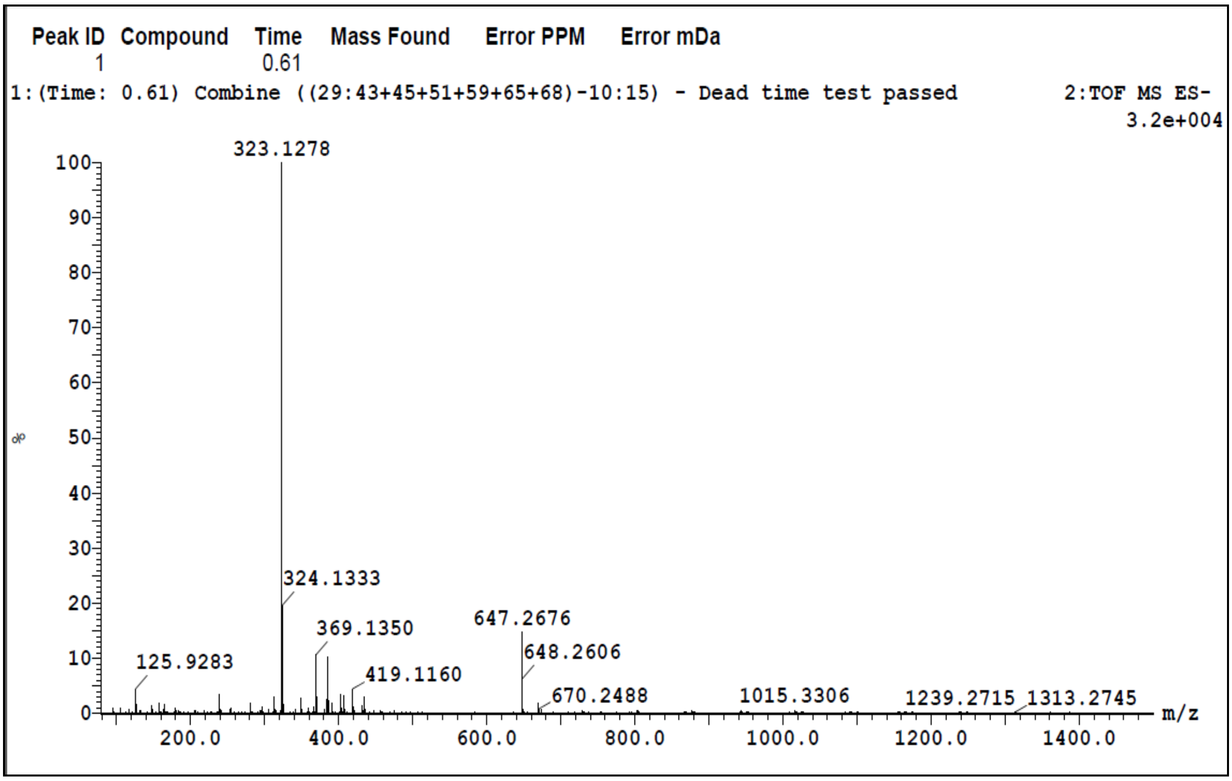

Figure S5. ^{1}H-NMR kingianic acid A (**1**) in $CDCl_3$ at 600 MHz.

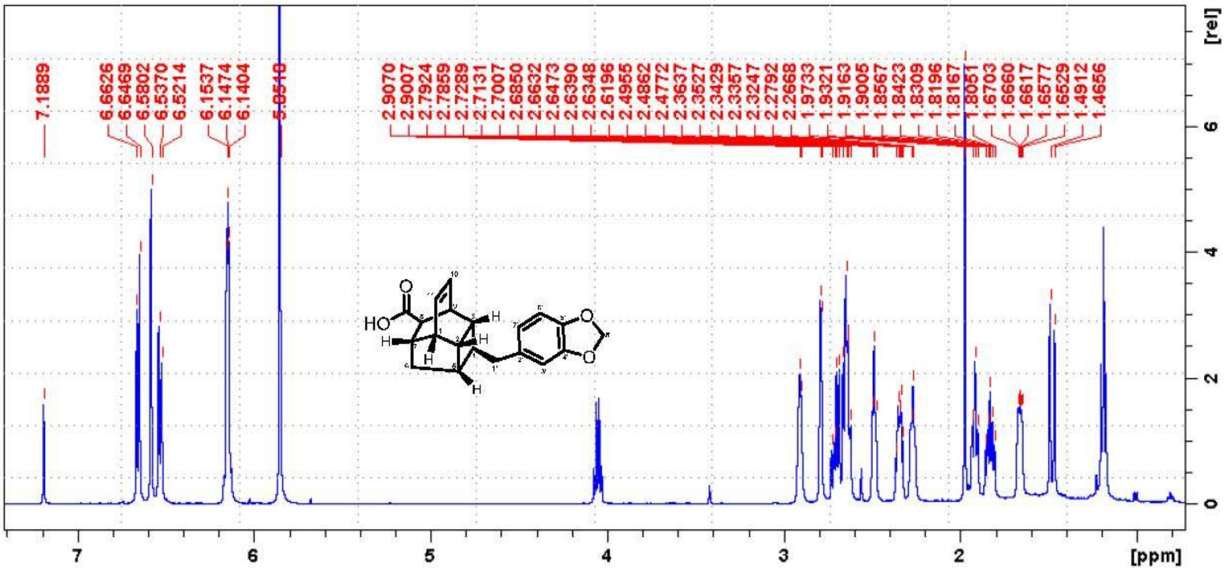

Figure S6. ^{13}C-NMR kingianic acid A (**1**) in $CDCl_3$ at 150 MHz.

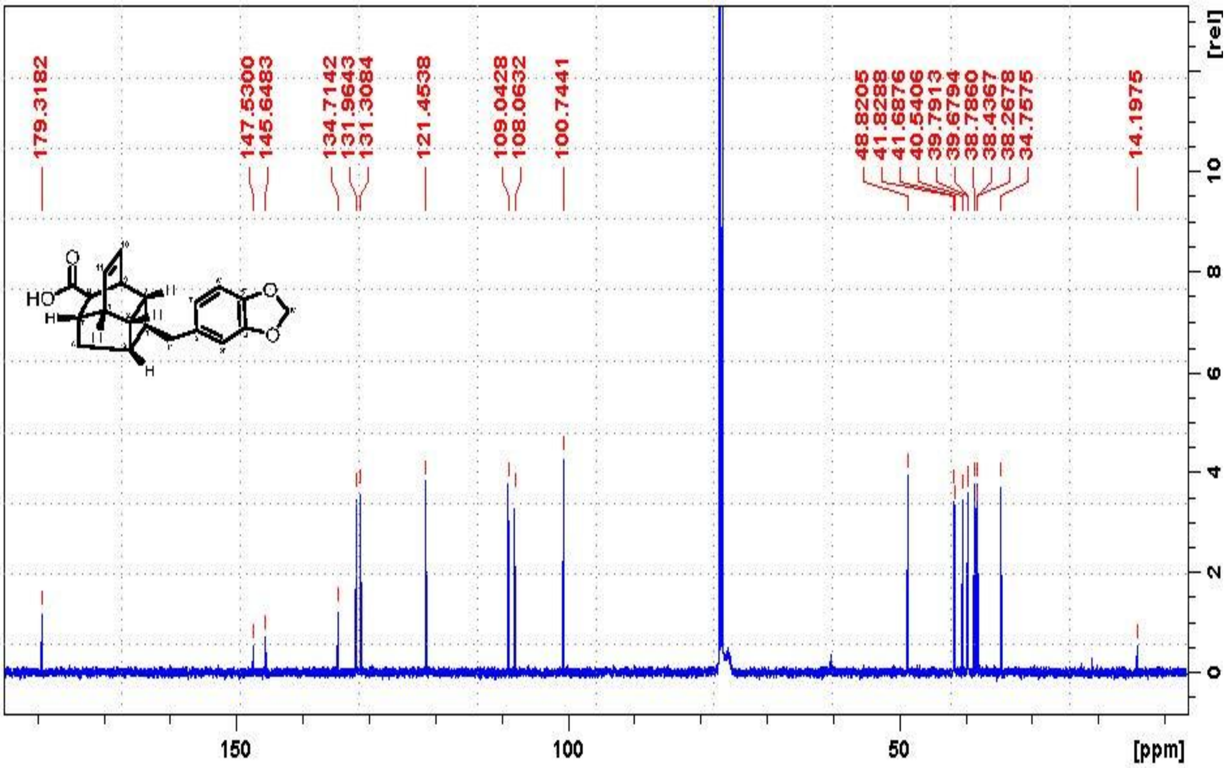

Figure S7. DEPT135 kingianic acid A (**1**) in $CDCl_3$.

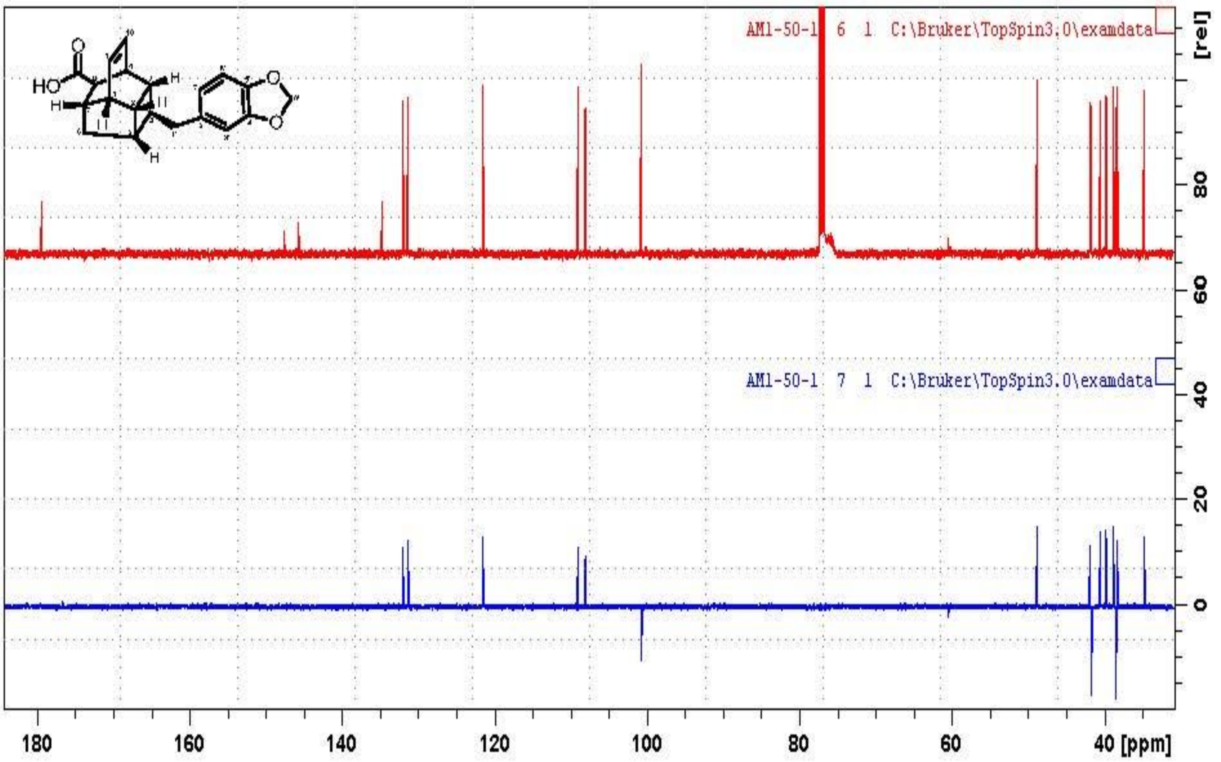

Figure S8. HRESIMS of kingianic acid B (**2**).

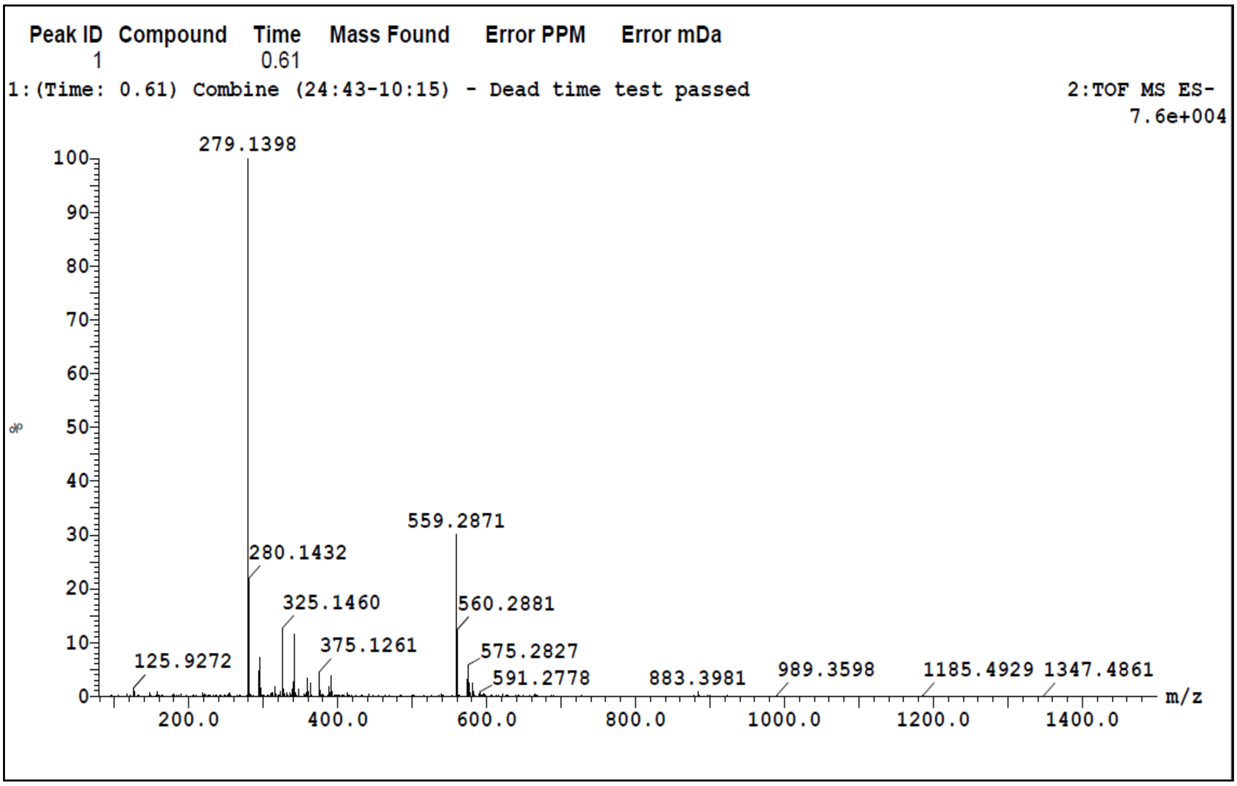

Figure S9. ^{1}H-NMR kingianic acid B (**2**) in $CDCl_3$ at 600 MHz.

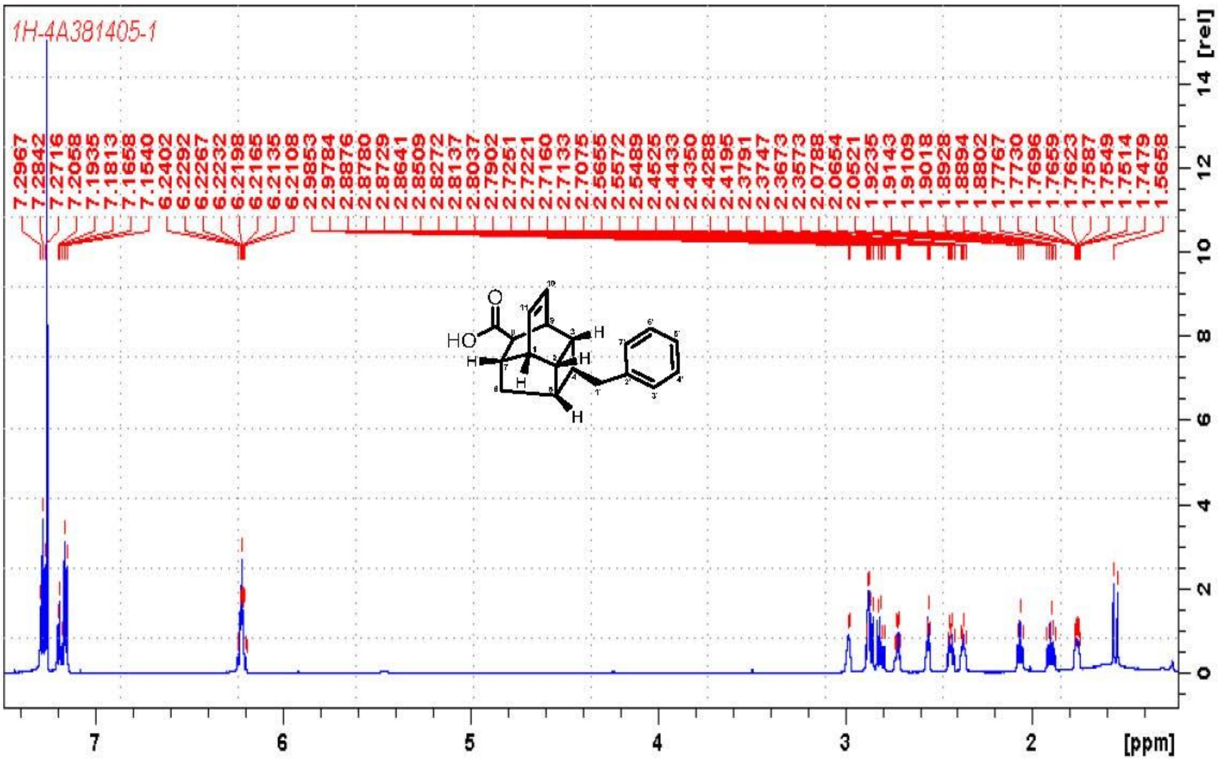

Figure S10. ^{13}C-NMR kingianic acid B (**2**) in $CDCl_3$ at 150 MHz.

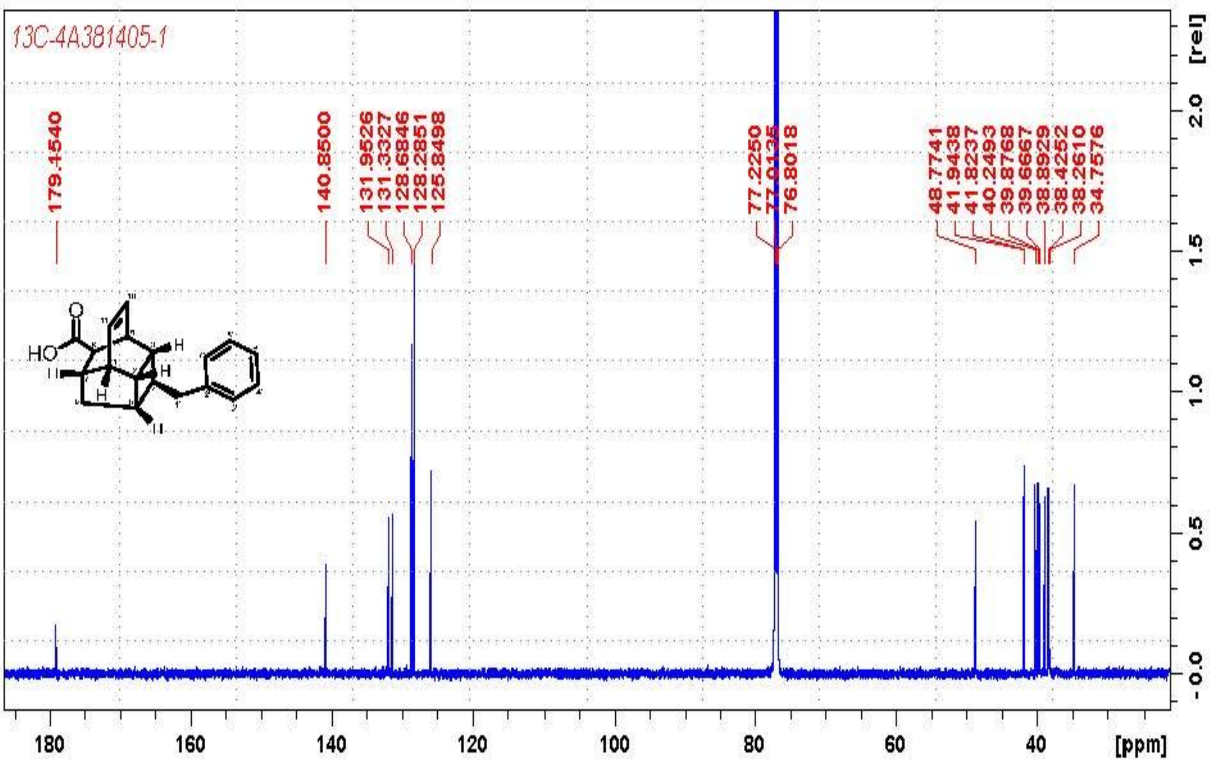

Figure S11. DEPT135 kingianic acid B (**2**) in $CDCl_3$.

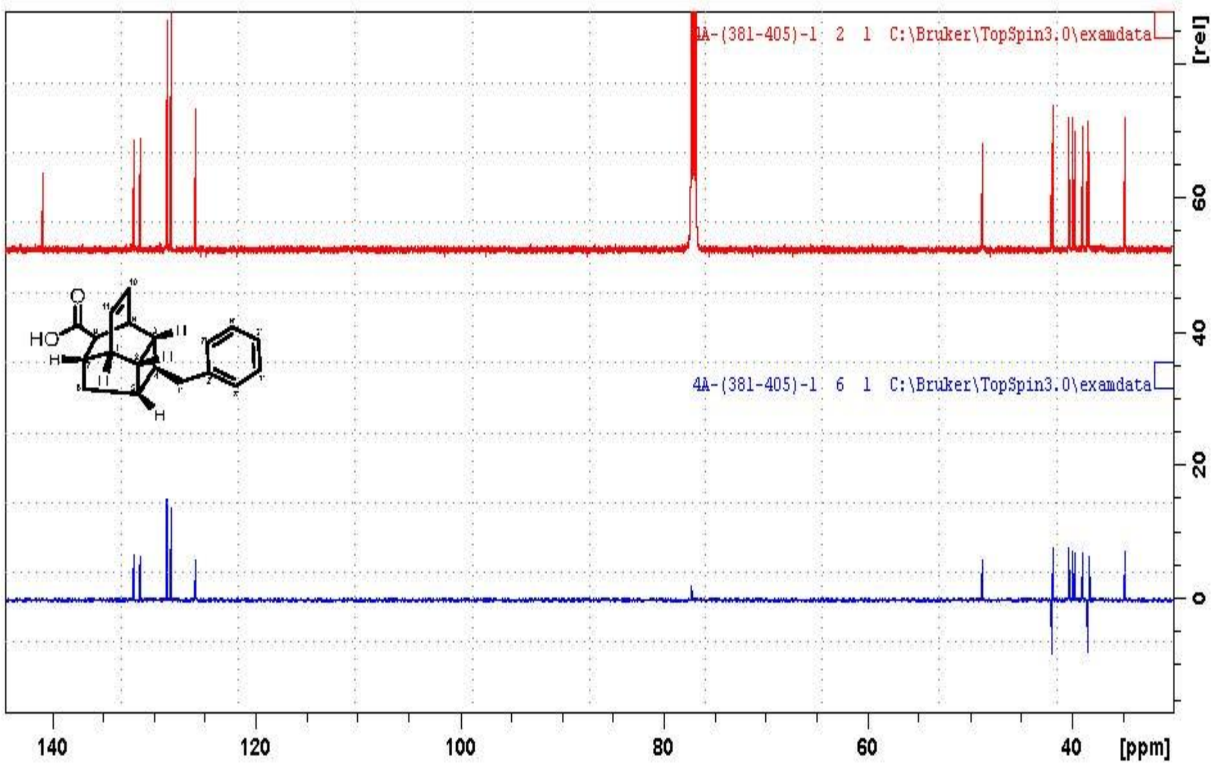

Figure S12. HRESIMS of kingianic acid C (**3**).

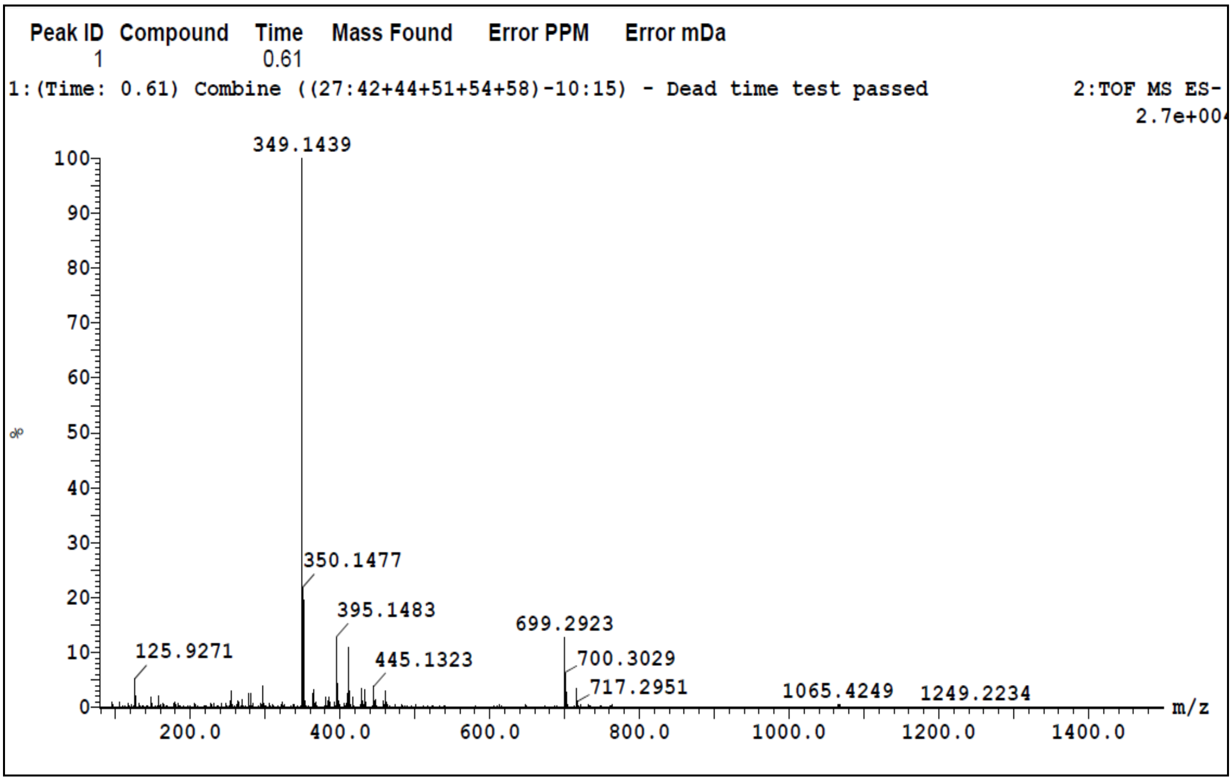

Figure S13. ^{1}H-NMR kingianic acid C (**3**) in $CDCl_3$ at 600 MHz.

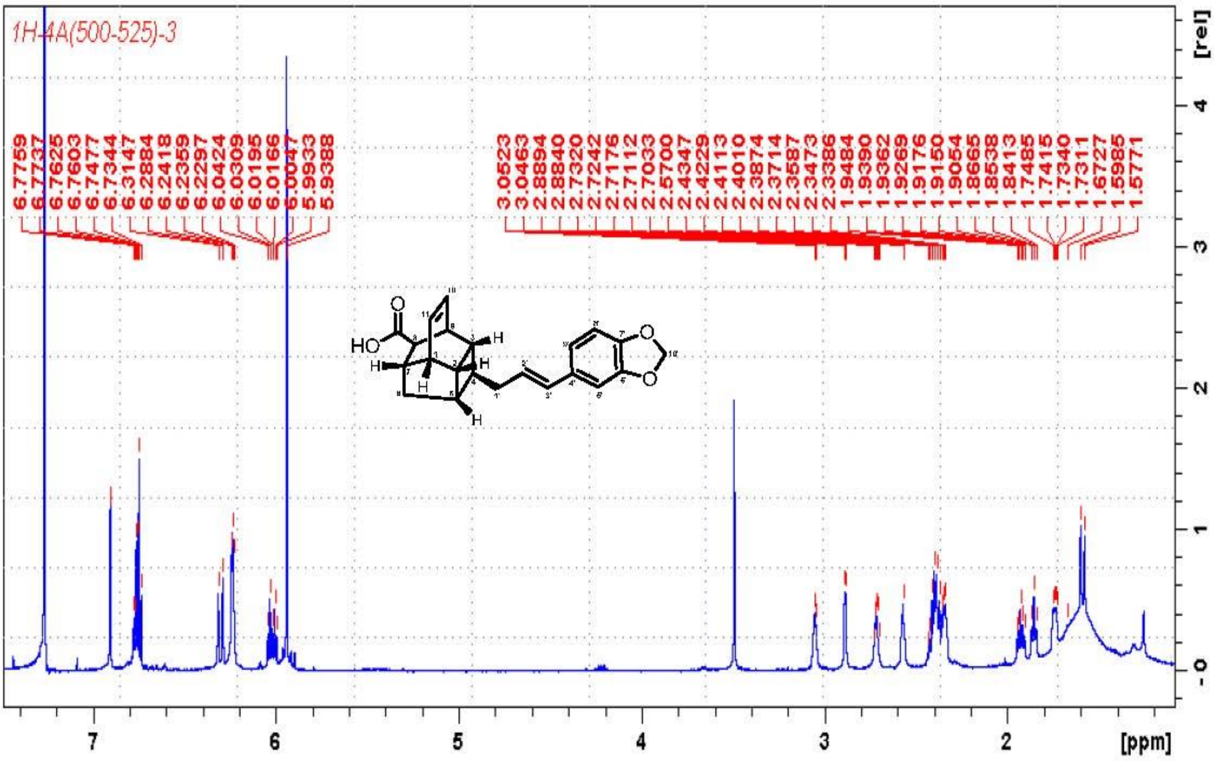

Figure S14. ^{13}C-NMR kingianic acid C (**3**) in $CDCl_3$ at 150 MHz.

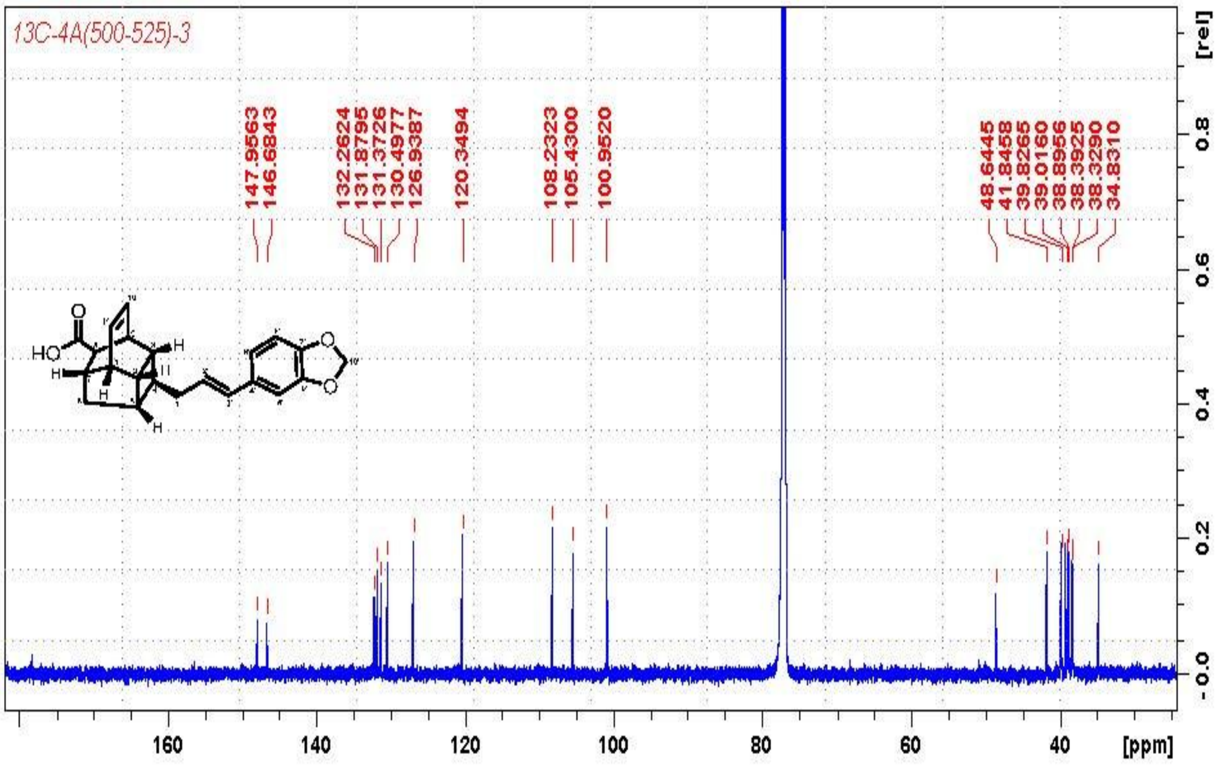

Figure S15. DEPT135 kingianic acid C (**3**) in $CDCl_3$.

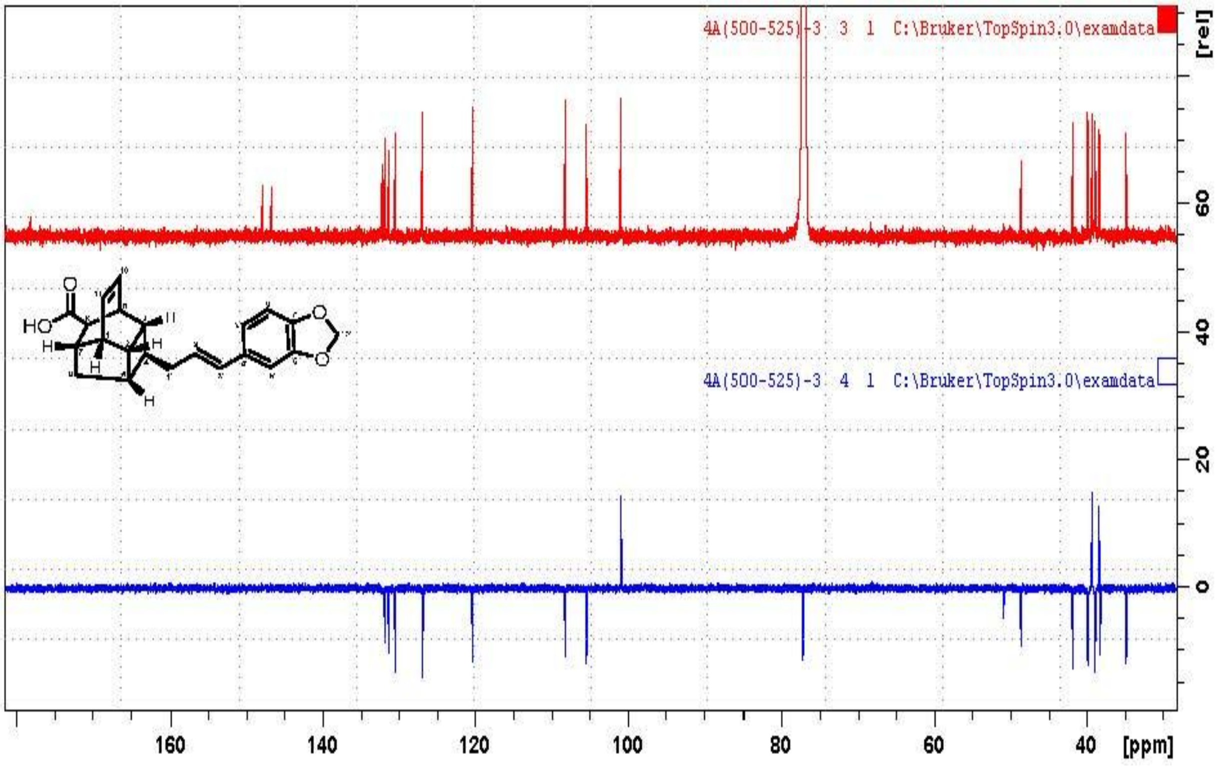

Figure S16. HRESIMS of kingianic acid D (**4**).

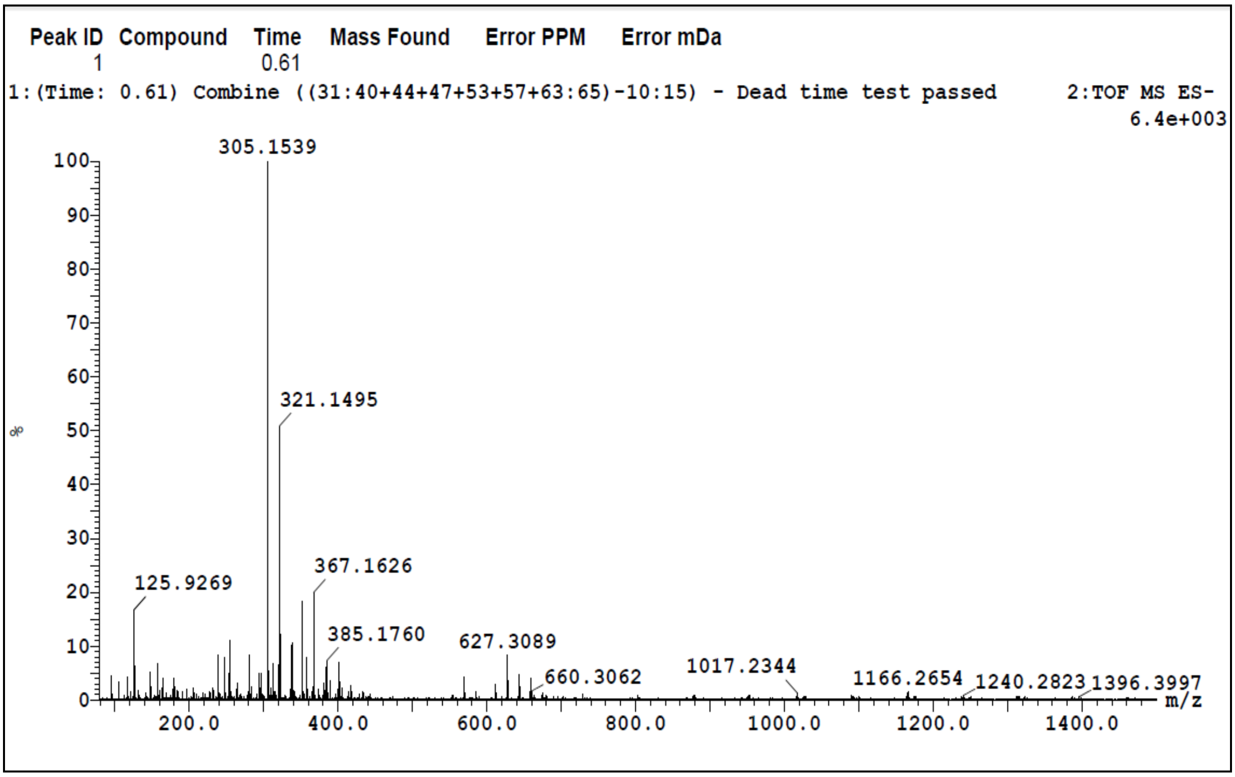

Figure S17. ^{1}H-NMR kingianic acid D (**4**) in $CDCl_3$ at 600 MHz.

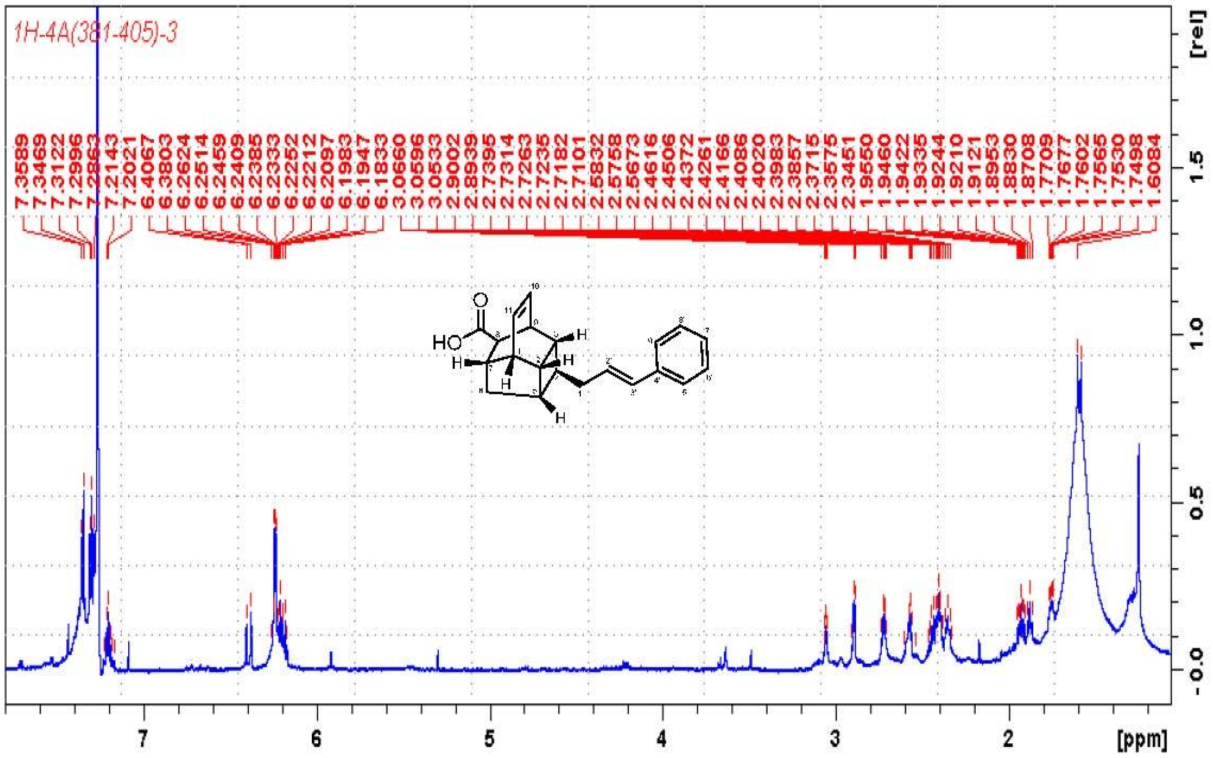

Figure S18. ^{13}C-NMR kingianic acid D (**4**) in $CDCl_3$ at 150 MHz.

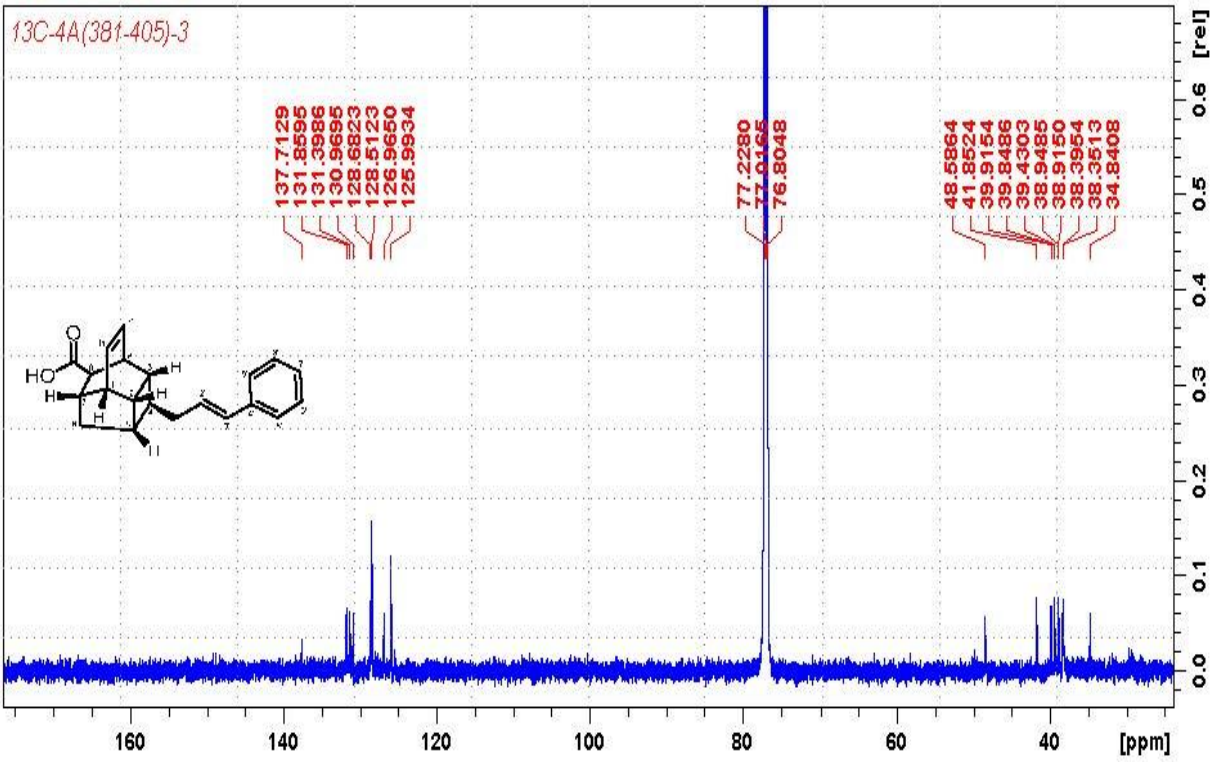

Figure S19. DEPT135 kingianic acid D (**4**) in $CDCl_3$.

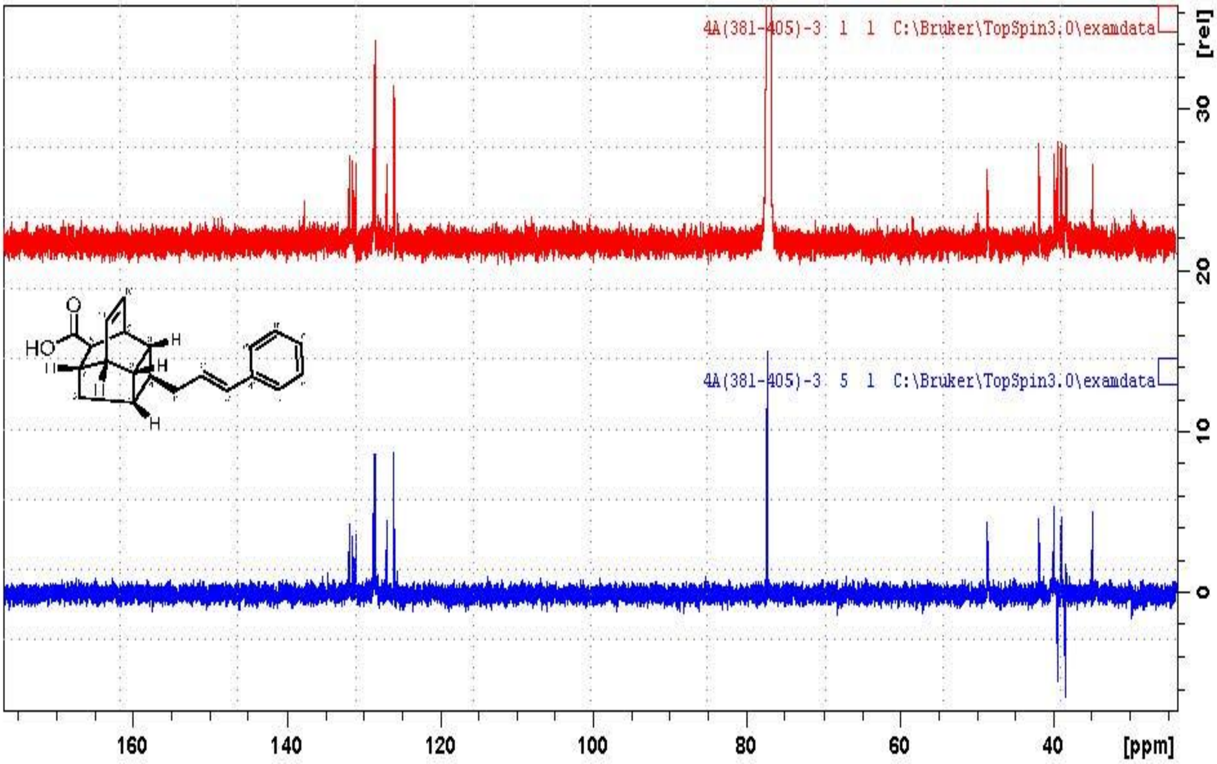

Figure S20. HRESIMS of kingianic acid E (**5**).

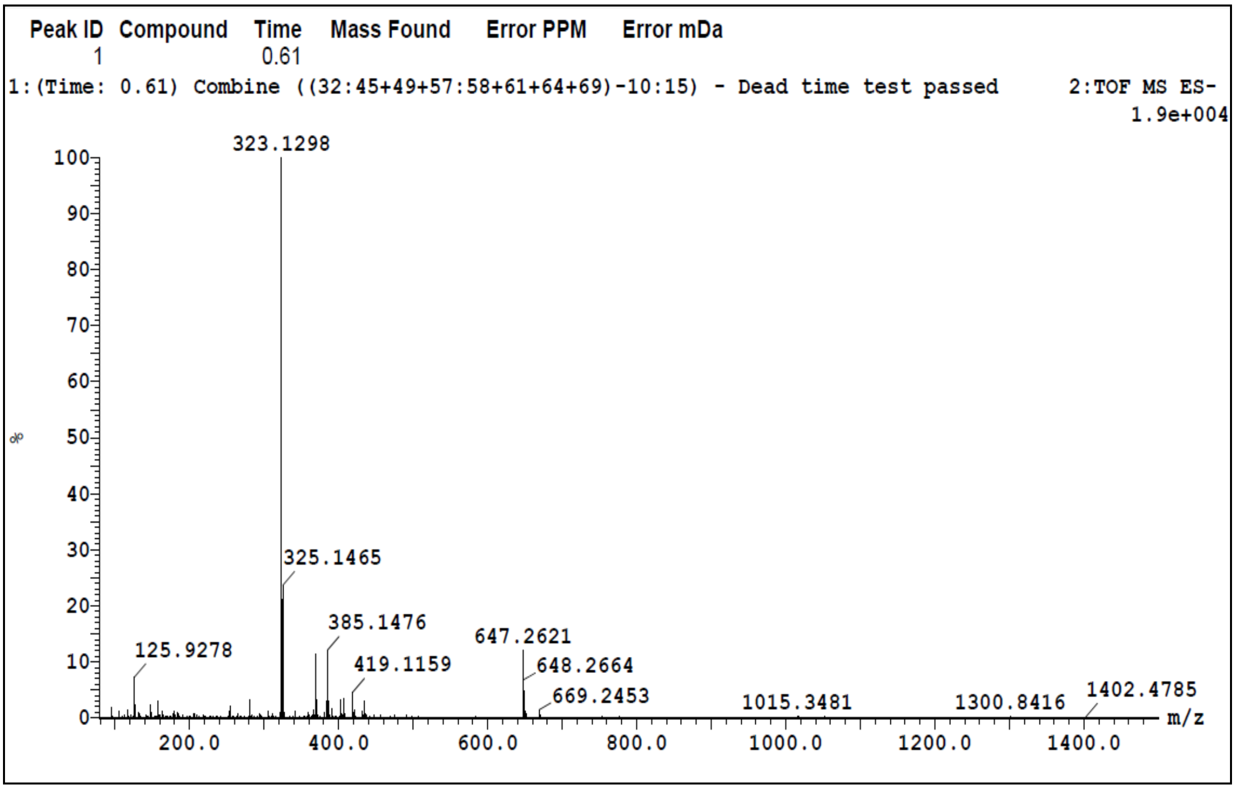

Figure S21. ^{1}H-NMR kingianic acid E (**5**) in $CDCl_3$ at 600 MHz.

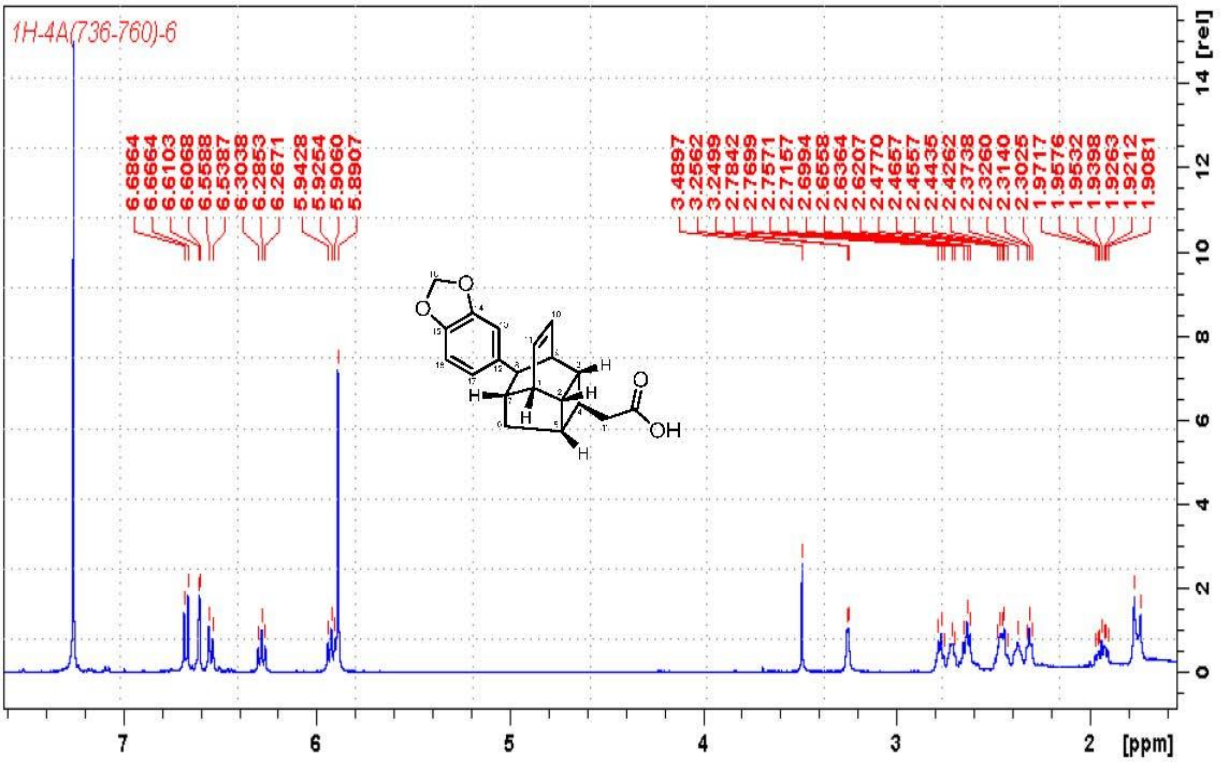

Figure S22. ^{13}C-NMR kingianic acid E (**5**) in $CDCl_3$ at 150 MHz.

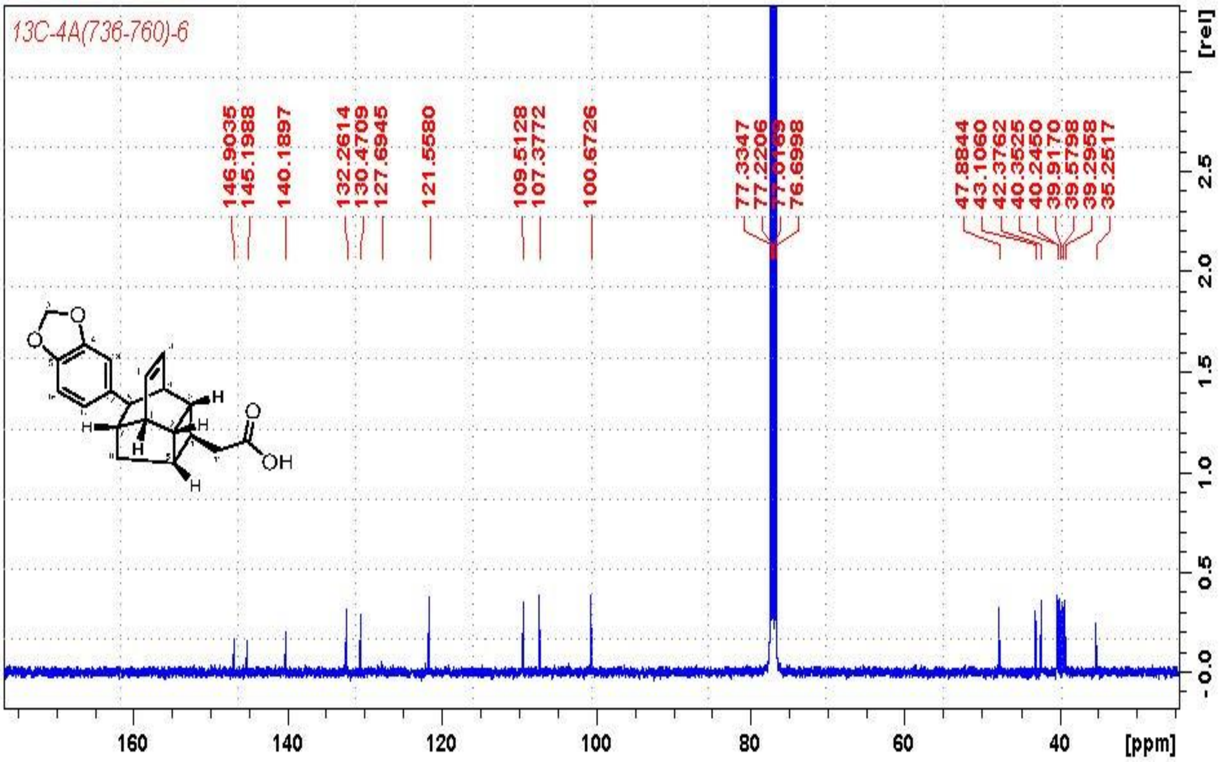

Figure S23. DEPT135 kingianic acid E (**5**) in $CDCl_3$.

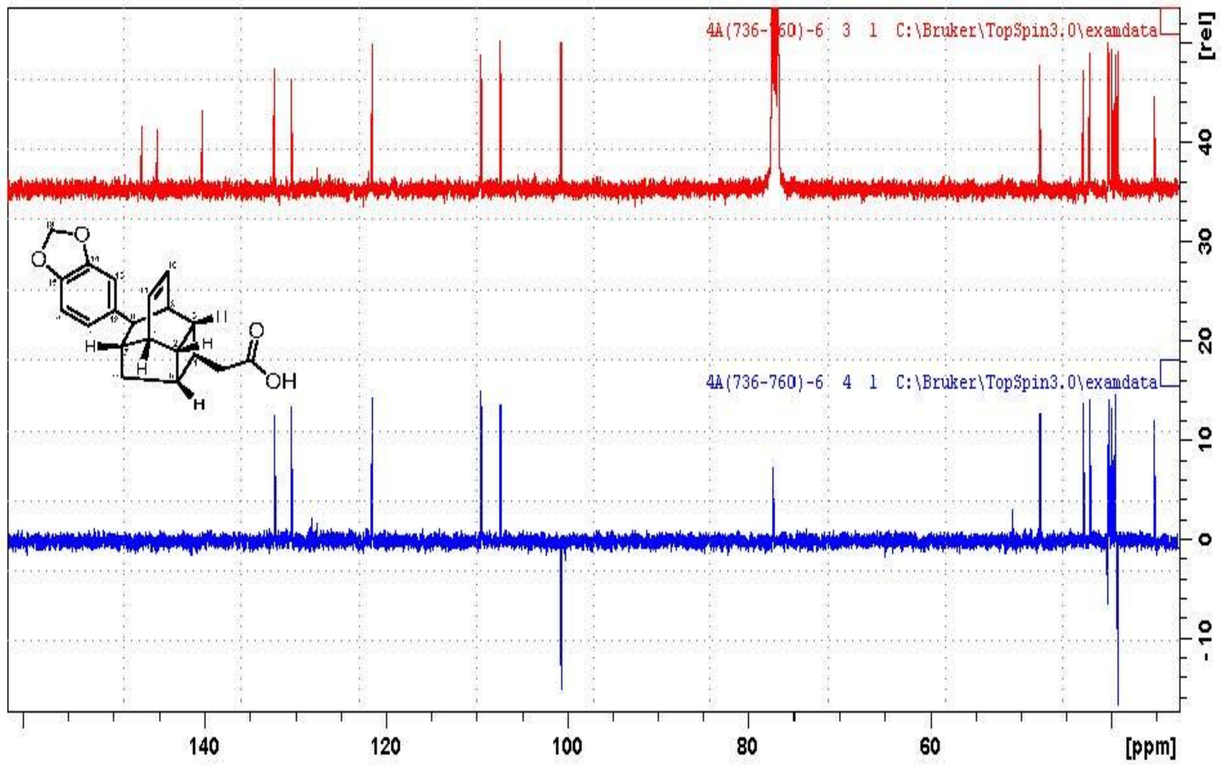

Figure S24. HRESIMS of kingianic acid F (**6**).

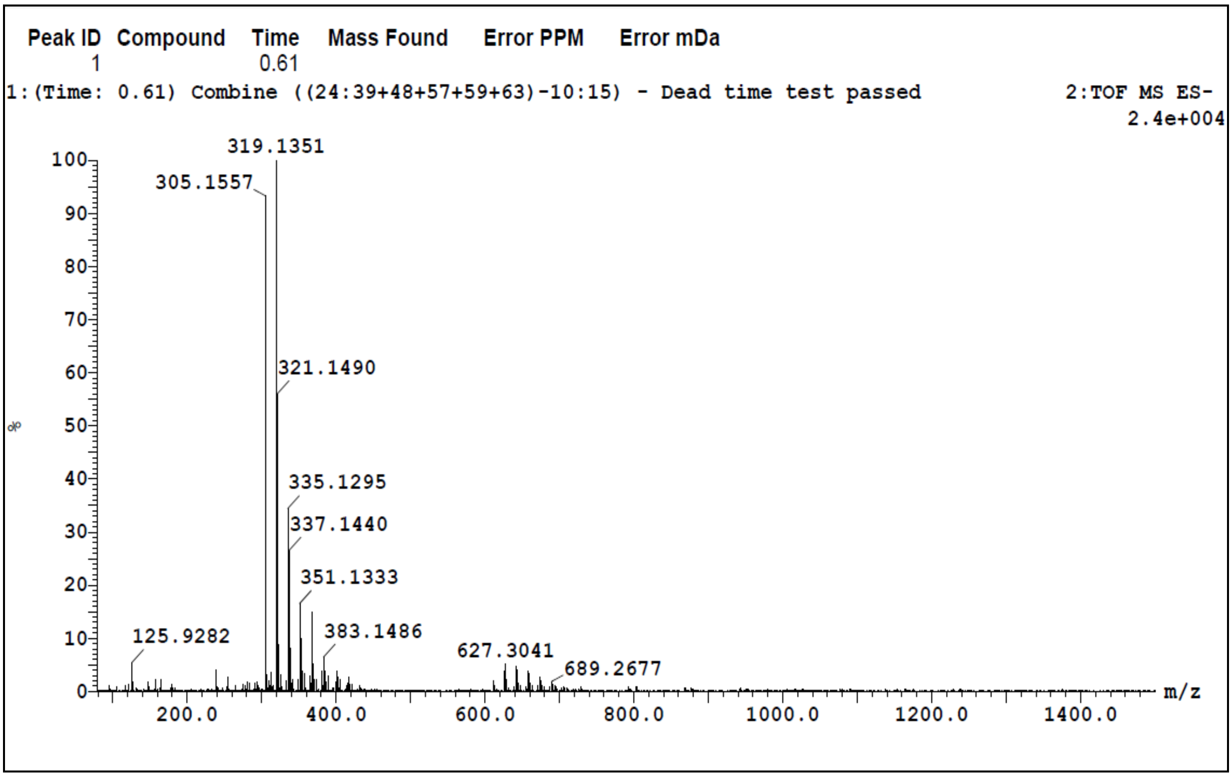

Figure S25. ^{1}H-NMR kingianic acid F (**6**) in $CDCl_3$ at 600 MHz.

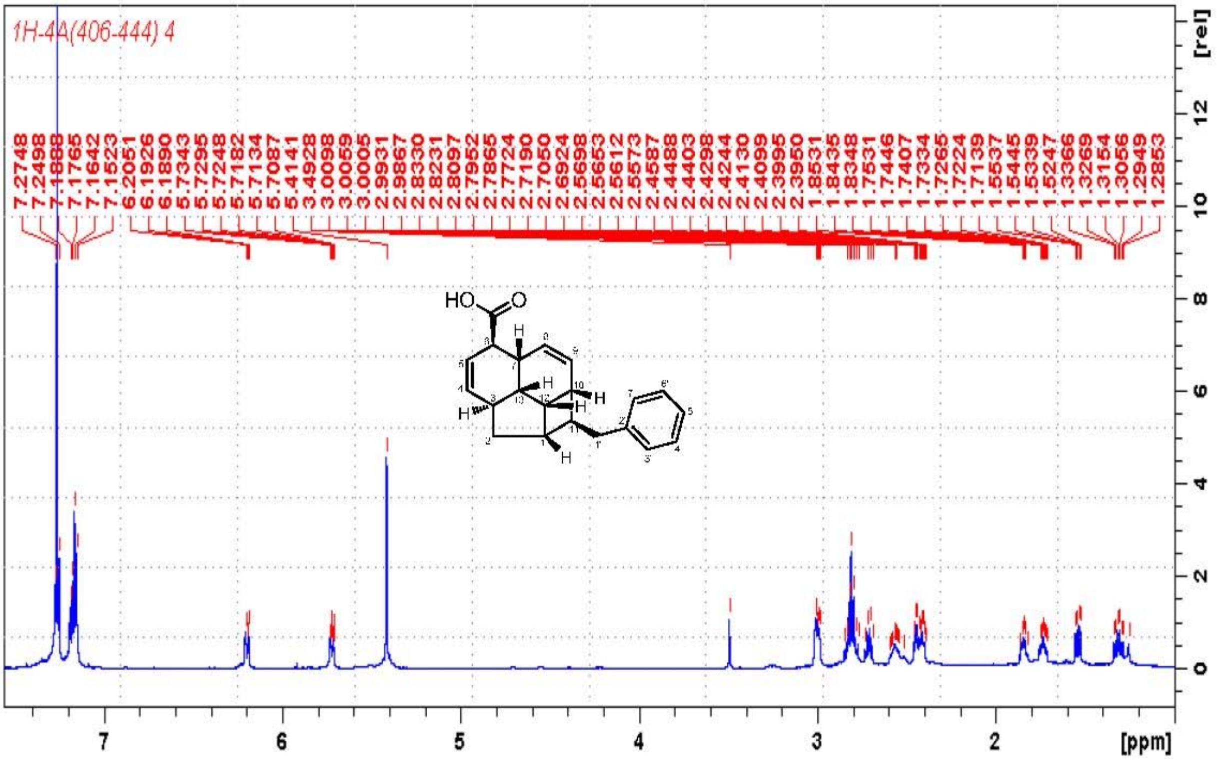

Figure S26. ^{13}C-NMR kingianic acid F (**6**) in $CDCl_3$ at 150 MHz.

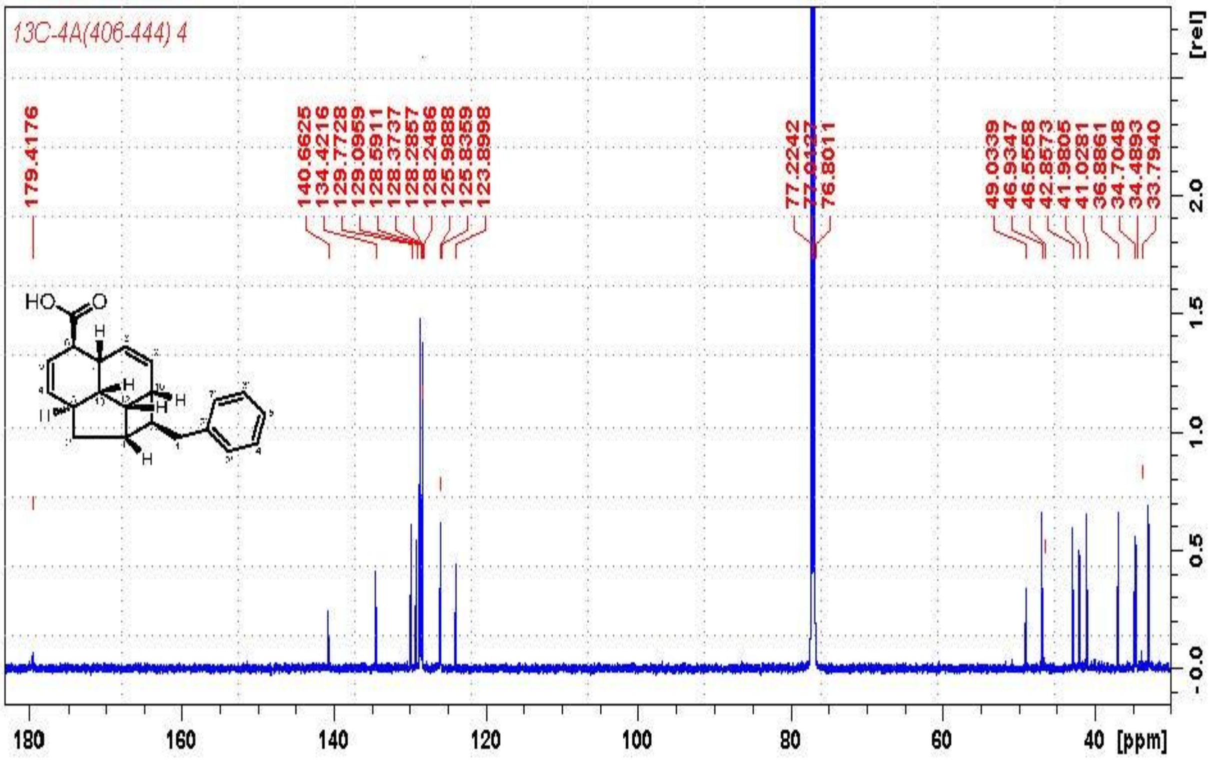

Figure S27. DEPT135 kingianic acid F (**6**) in $CDCl_3$.

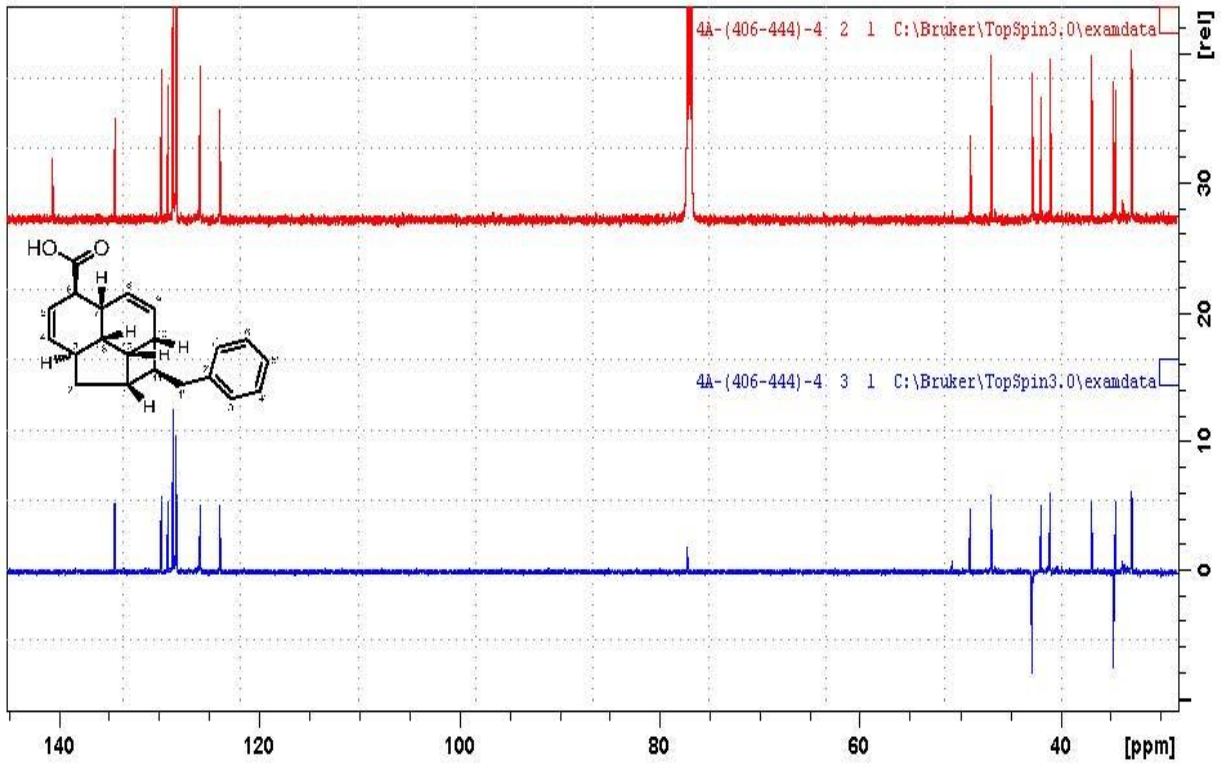

Figure S28. HRESIMS of kingianic acid G (**7**).

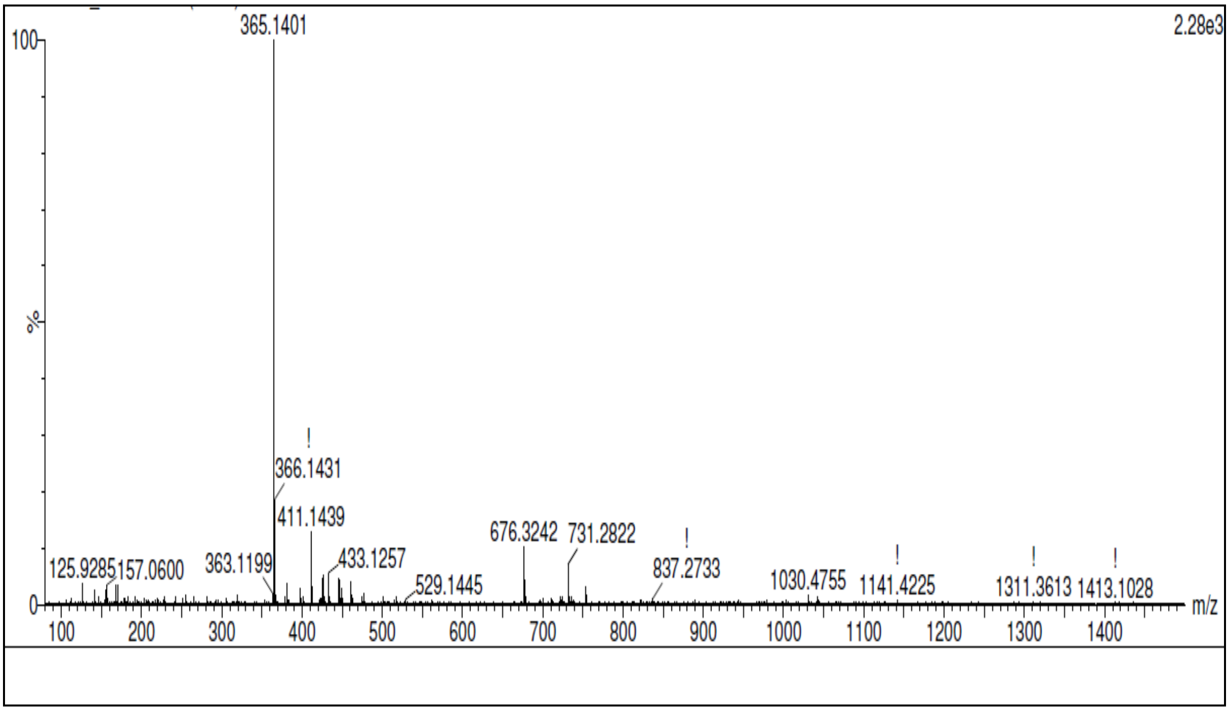

Figure S29. ^{1}H-NMR kingianic acid G (**7**) in $CDCl_3$ at 600 MHz.

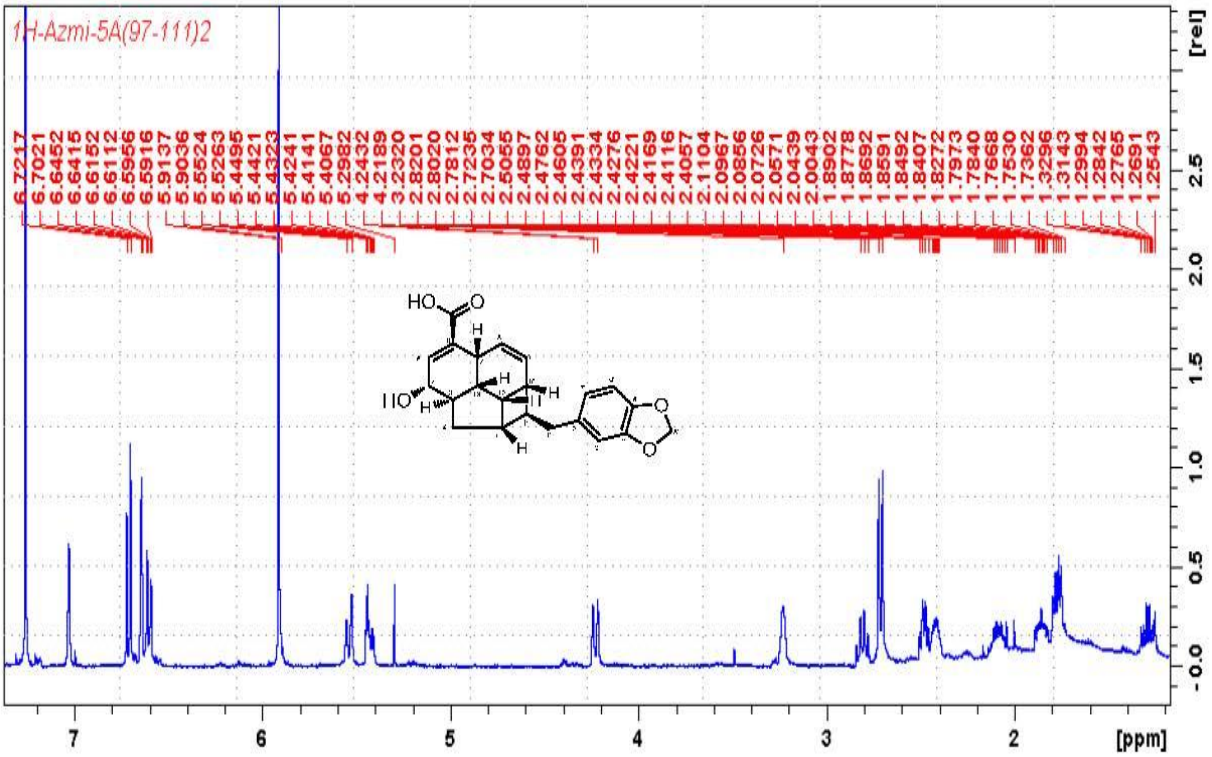

Figure S30. ^{13}C-NMR kingianic acid G (**7**) in $CDCl_3$ at 150 MHz.

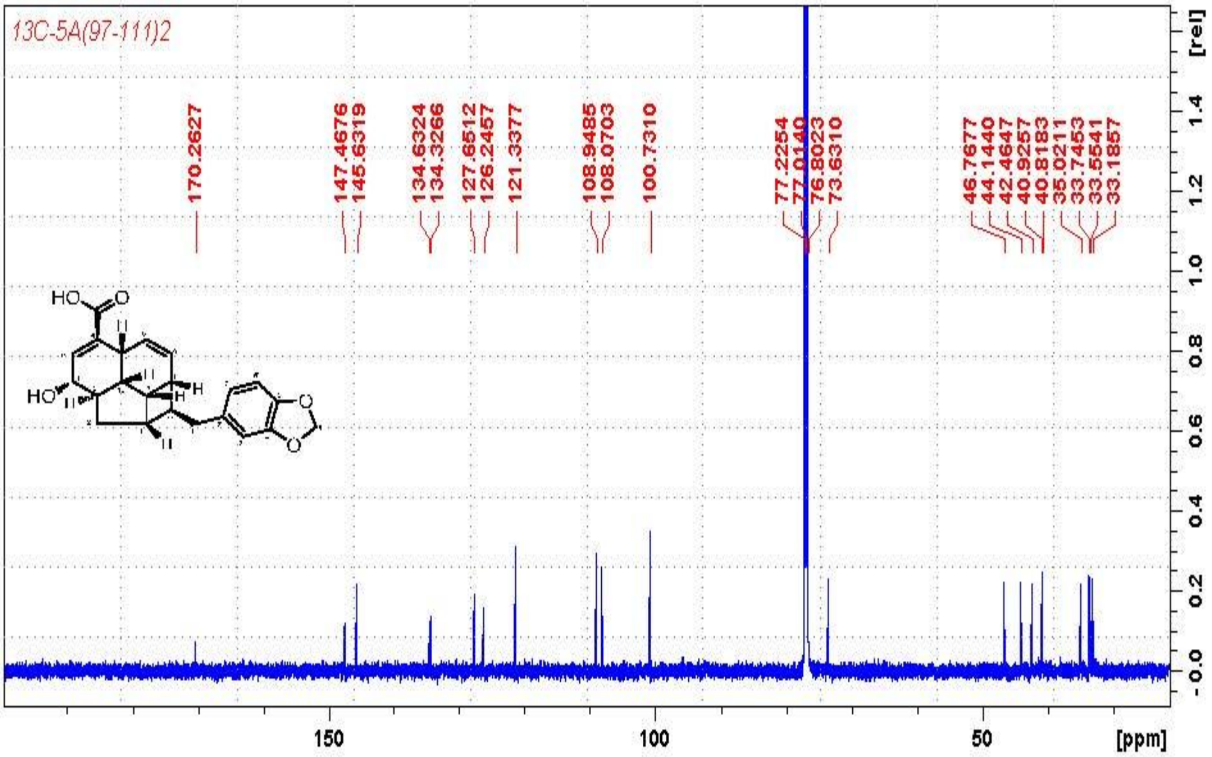

Figure S31. DEPT135 kingianic acid G (**7**) in $CDCl_3$.

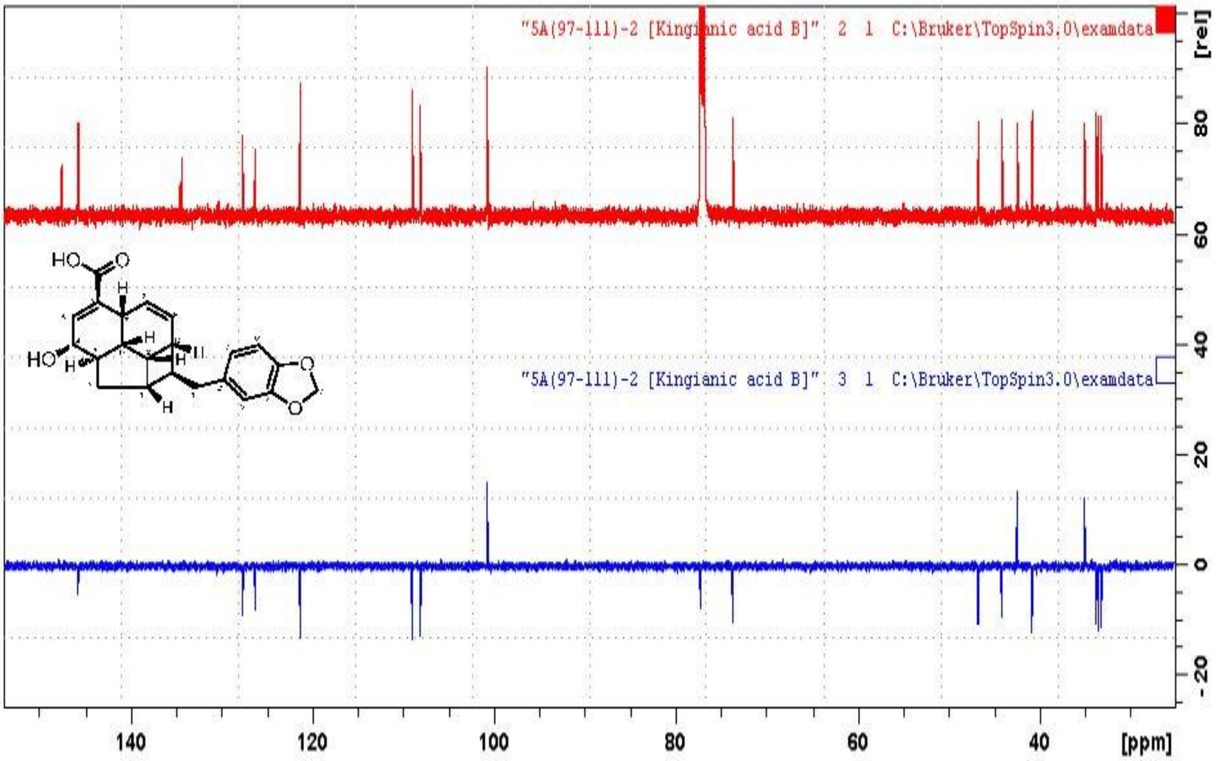

Figure S32. HRESIMS of endiandric acid M (**8**).

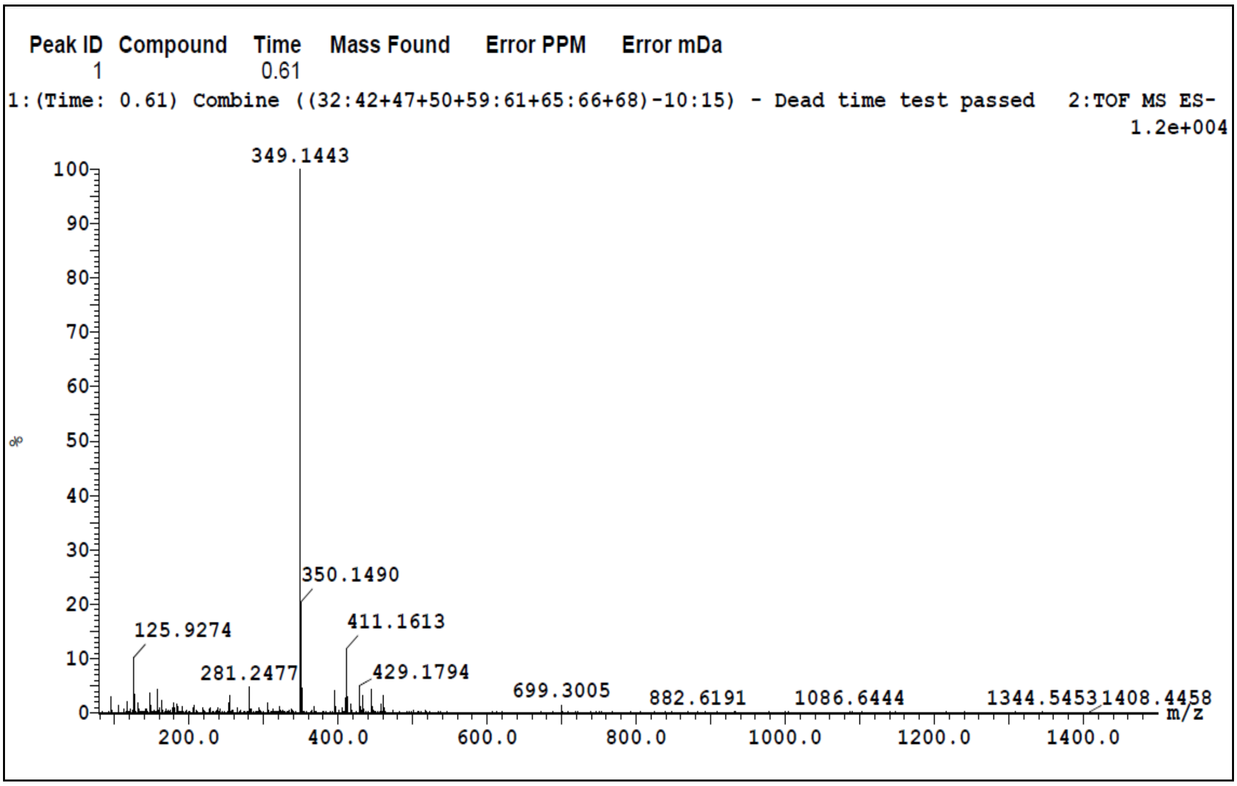

Figure S33. ^{1}H-NMR endiandric acid M (**8**) in $CDCl_3$ at 600 MHz.

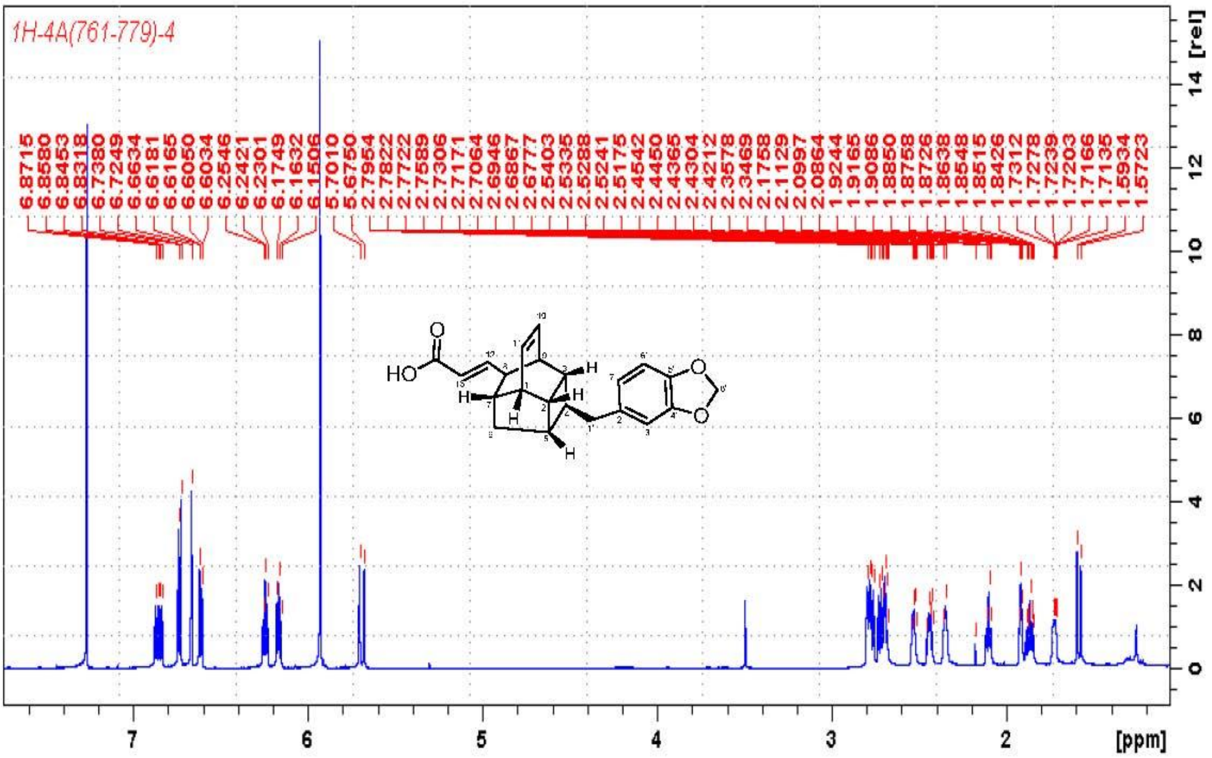

Figure S34. ^{13}C-NMR endiandric acid M (**8**) in $CDCl_3$ at 150 MHz.

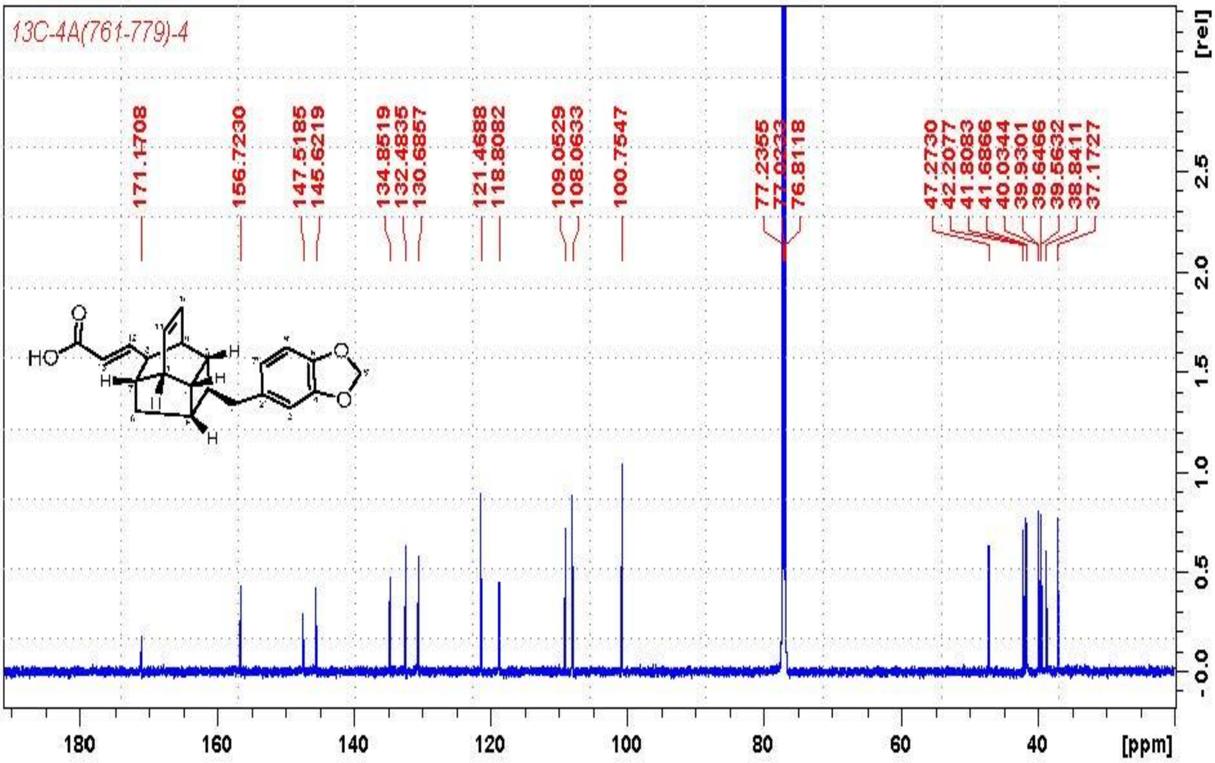

Figure S35. DEPT135 endiandric acid M (**8**) in $CDCl_3$.

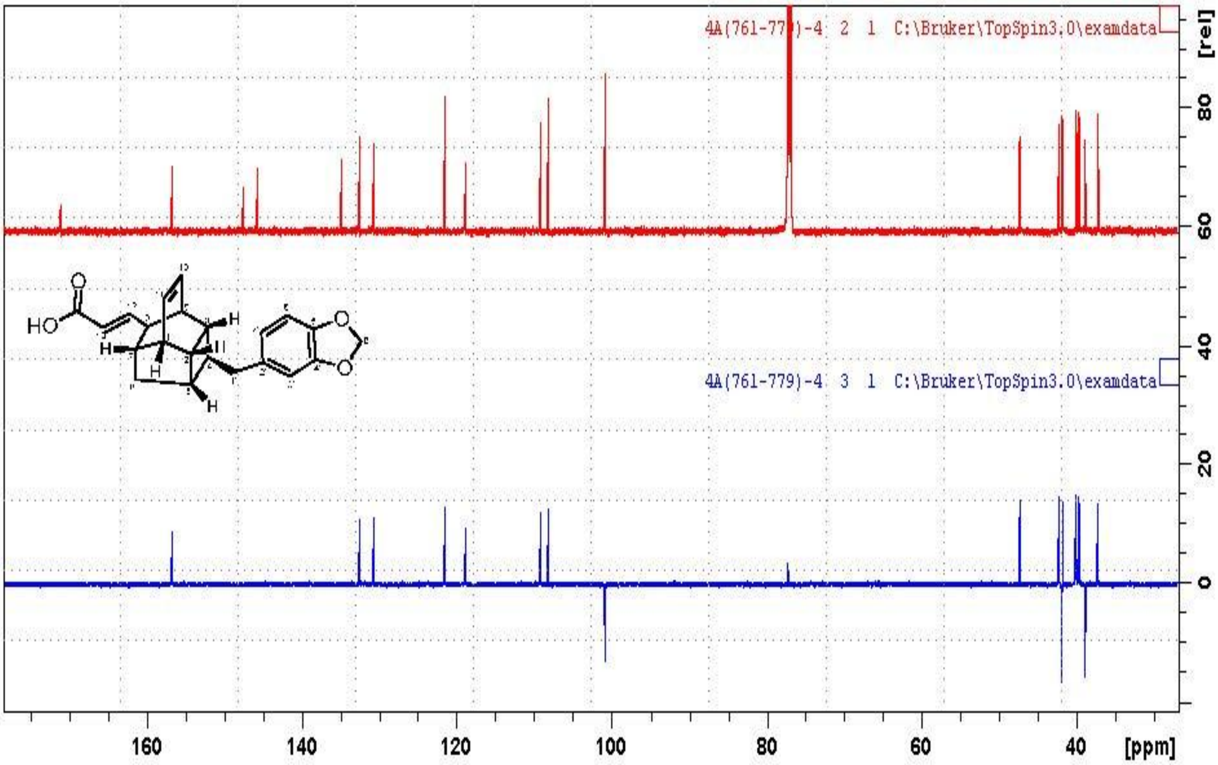

Figure S36. HRESIMS of endiandric acid **10**.

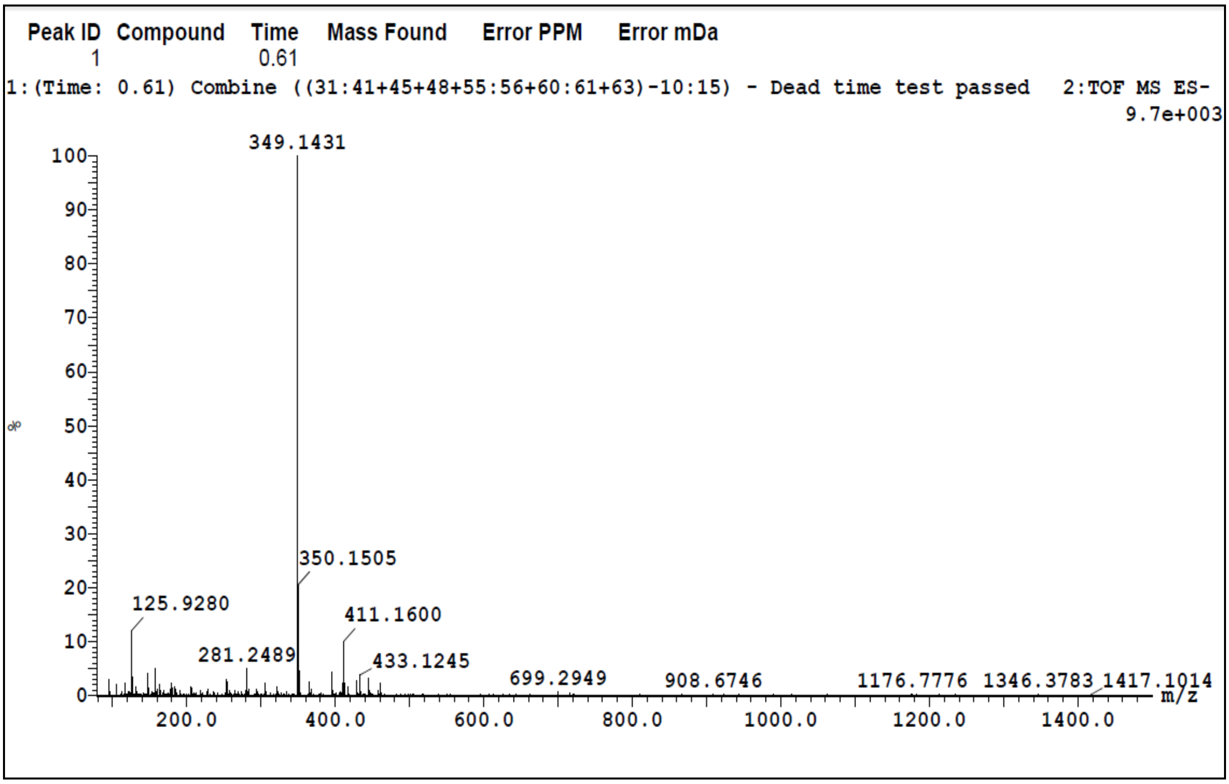

Figure S37. ^{1}H-NMR endiandric acid **10** in $CDCl_3$ at 600 MHz.

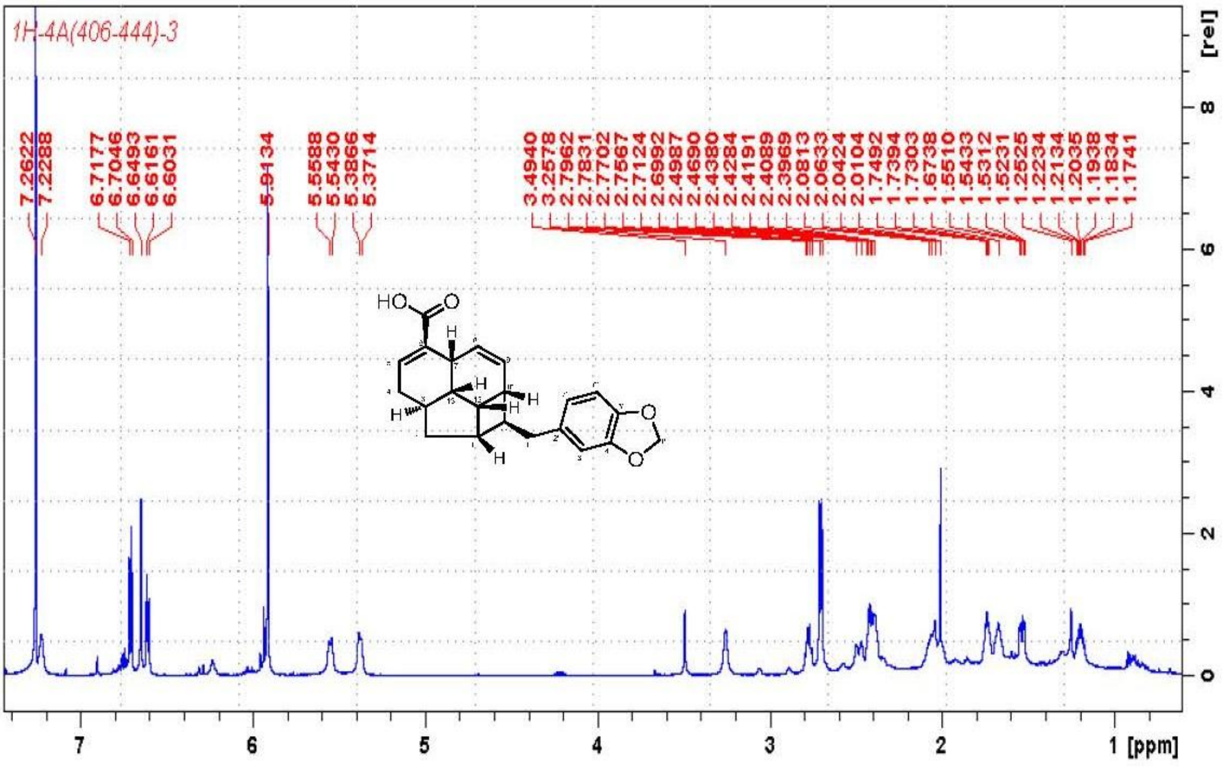

Figure S38. ^{13}C-NMR endiandric acid **10** in $CDCl_3$ at 150 MHz.

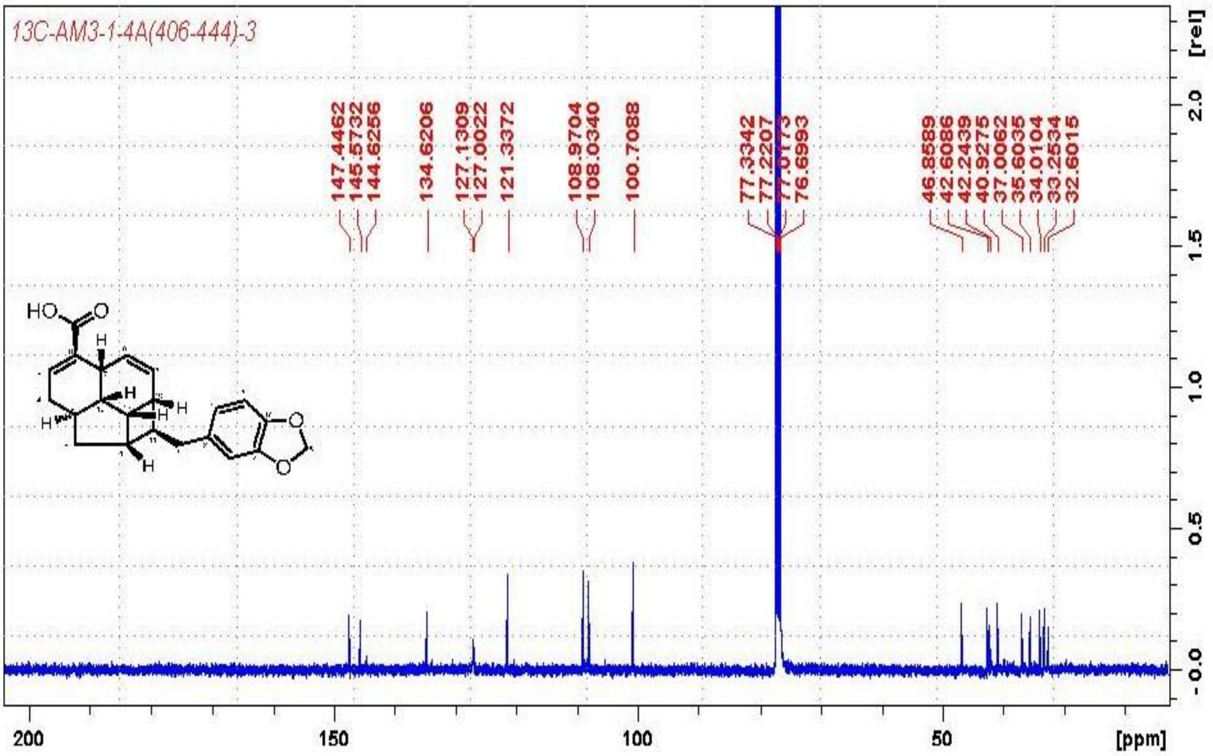

Figure S39. DEPT135 endiandric acid **10** in $CDCl_3$.

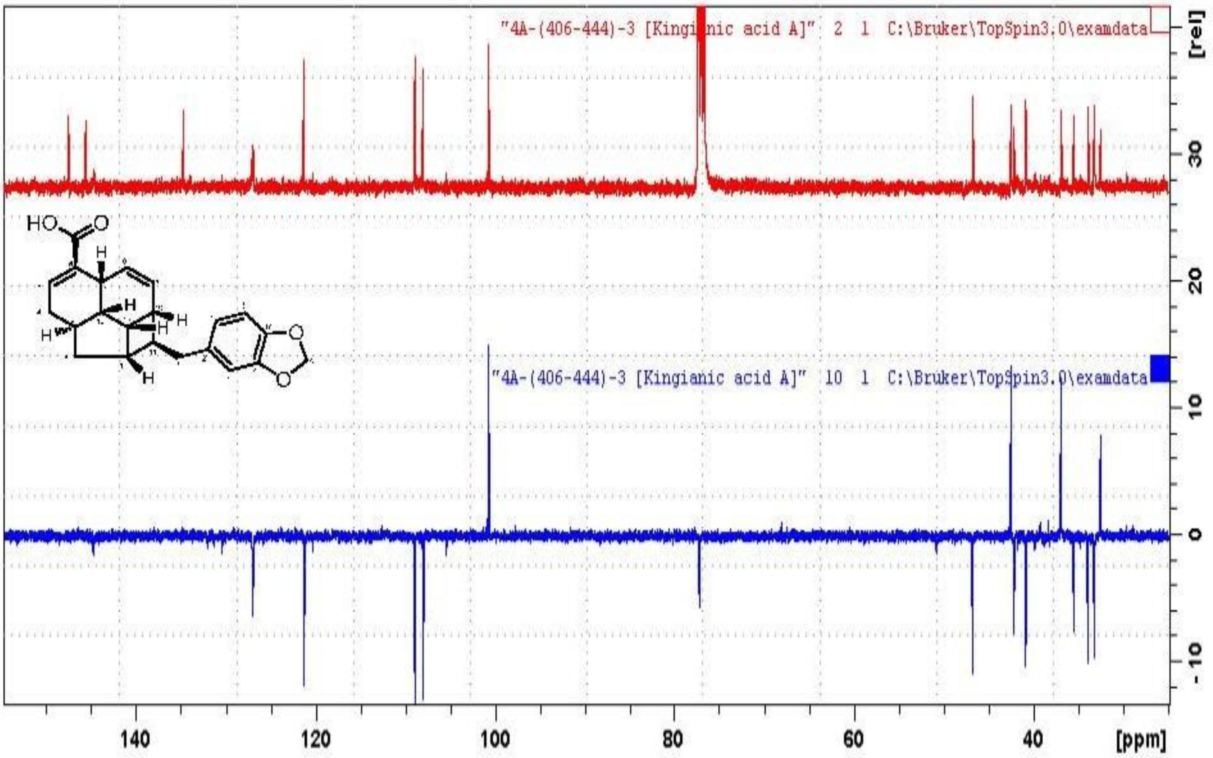

Sequences alignment of Bcl-xL and Mcl-1 with Bak-BH3 and Bid-BH3 peptides, respectively.

Both protein targets include an N-terminal His-tag followed by the thrombin cleavage site, to facilitate purification. Our Bcl-xL construct includes the four homology domains of the human protein, whereas our Mcl-1 construct includes the three homology domains of the mouse protein. Trans-membrane domains have been deleted in both sequences.

**Bak (PolyP) →**	**---------GQ-- 2**
**Bcl-xL(ICSN)→**	**MHHHHHHSSGLVPRGSEFMSQSNRELVVDFLSYKLSQKGYSWSQFSDVEENRTE APEGTE 60**
**Bak (PolyP) →**	**---VGRQLAIIGDDINR-- 16**
**Bcl-xL(ICSN)→**	**SEAVKQALREAGDEFELRYRRAFSDLTSQLHITPGTAYQSFEQVVNELFRDGVNW GRIVA 120**
**Bak (PolyP) →**	**--**
**Bcl-xL(ICSN)→**	**FFSFGGALCVESVYKEMQVLVSRIAAWMATYLNDHLEPWIQENGGWDTFVELYG 174**

→ BH4 domain
→ BH3 domain
→ BH1 domain
→ BH2 domain

**Bid (PolyP) →**	**---------------------EDII------------------------------RNI 7**
**Mcl-1 (ICSN)→**	**MGSSHHHHHHSSGLVPRGSHMEDDLYRQSLEIISRYLREQATGSKDSKPLGEAGA AGRRA 60**
**Bid (PolyP) →**	**ARHLAQVGDSMDR-- 20**
**Mcl-1 (ICSN)→**	**LETLRRVGDGVQRNHETAFQGMLRKLDIKNEGDVKSFSRVMVHVFKDGVTNWG RIVTLIS 120**
**Bid (PolyP) →**	**---**
**Mcl-1 (ICSN)→**	**FGAFVAKHLKSVNQESFIEPLAETITDVLVRTKRDWLVKQRGWDGFVEFFHVQD LE 176**

→ BH3 domain
→ BH1 domain
→ BH2 domain
